# A Model of Cardiovascular Disease Giving a Plausible Mechanism for the Effect of Fractionated Low-Dose Ionizing Radiation Exposure

**DOI:** 10.1371/journal.pcbi.1000539

**Published:** 2009-10-23

**Authors:** Mark P. Little, Anna Gola, Ioanna Tzoulaki

**Affiliations:** Department of Epidemiology and Public Health, Faculty of Medicine, Imperial College London, London, United Kingdom; McMaster University, Canada

## Abstract

Atherosclerosis is the main cause of coronary heart disease and stroke, the two major causes of death in developed society. There is emerging evidence of excess risk of cardiovascular disease at low radiation doses in various occupationally exposed groups receiving small daily radiation doses. Assuming that they are causal, the mechanisms for effects of chronic fractionated radiation exposures on cardiovascular disease are unclear. We outline a spatial reaction-diffusion model for atherosclerosis and perform stability analysis, based wherever possible on human data. We show that a predicted consequence of multiple small radiation doses is to cause mean chemo-attractant (MCP-1) concentration to increase linearly with cumulative dose. The main driver for the increase in MCP-1 is monocyte death, and consequent reduction in MCP-1 degradation. The radiation-induced risks predicted by the model are quantitatively consistent with those observed in a number of occupationally-exposed groups. The changes in equilibrium MCP-1 concentrations with low density lipoprotein cholesterol concentration are also consistent with experimental and epidemiologic data. This proposed mechanism would be experimentally testable. If true, it also has substantive implications for radiological protection, which at present does not take cardiovascular disease into account. The Japanese A-bomb survivor data implies that cardiovascular disease and cancer mortality contribute similarly to radiogenic risk. The major uncertainty in assessing the low-dose risk of cardiovascular disease is the shape of the dose response relationship, which is unclear in the Japanese data. The analysis of the present paper suggests that linear extrapolation would be appropriate for this endpoint.

## Author Summary

Atherosclerosis is the main cause of coronary heart disease and stroke, the two major causes of death in developed society. There is emerging evidence of excess risk of cardiovascular disease in various occupationally exposed groups, exposed to fractionated radiation doses with small doses/fraction. The mechanisms for such effects of fractionated low-dose radiation exposures on cardiovascular disease are unclear. We outline a spatial reaction-diffusion model for early stage atherosclerotic lesion formation and perform a stability analysis, based on experimentally derived parameters. We show that following multiple small radiation doses the chemo-attractant (MCP-1) concentration increases proportionally to cumulative dose; this is driven by radiation-induced monocyte death. This will result in risk of atherosclerosis increasing approximately linearly with cumulative dose. This proposed mechanism would be testable. If true, it also has substantive implications for radiological protection, which at present does not take cardiovascular disease into account. The major uncertainty in assessing low-dose risk of cardiovascular disease is the shape of the dose response relationship, which is unclear in high dose data. Our analysis suggests that linear extrapolation would be appropriate.

## Introduction

Atherosclerosis is the main cause of coronary heart disease and stroke, the two major causes of death in developed society [1]. Though previously initiation of atherosclerosis was attributed mainly to lipid accumulation within the arterial walls, it is now widely accepted that inflammation plays a vital role in the initiation and progression of the disease [2]–[5].

For some time cardiovascular effects of high dose radiotherapy (RT) have been known [6],[7]. A variety of effects are observed, presumed to result from inactivation of large numbers of cells and associated functional impairment of the affected tissue. Among such effects are direct damage to the structures of the heart – including marked diffuse fibrotic damage, especially of the pericardium and myocardium, pericardial adhesions, microvascular damage and stenosis of the valves − and to the coronary arteries; these sorts of damage occur both in patients receiving RT and in experimental animals [6]. There is emerging evidence of excess risk of cardiovascular disease at much lower radiation doses and occurring a long time after radiation exposure in the Japanese atomic bomb survivor Life Span Study (LSS) cohort [8],[9] and in various occupationally-exposed groups [10]–[14] although not in all (e.g., [15]). Assuming that they are causal, the likely mechanisms for such effects of low dose and/or chronic radiation exposures on cardiovascular disease are not clear [16],[17]. It is of interest that elevated levels of the pro-inflammatory cytokines IL-6, CRP, TNF-α and INF-γ, but also increased levels of the (generally) anti-inflammatory cytokine IL-10, have been observed in the Japanese atomic bomb survivors [18],[19]. There was also dose-related elevation in erythrocyte sedimentation rate and in levels of IgG, IgA and total immunoglobulins in this cohort, all markers of systemic inflammation [19].

In this paper we outline a mathematical formulation of a model of cardiovascular disease that is largely based on the inflammatory hypothesis articulated by Ross [2],[3]. The motivation behind the mathematical modelling is to encompass various factors contributing to the inflammatory process and subsequently to atherosclerotic formation. As atherosclerosis is not only a multifactorial, but also a multi-step disease, we concentrate on modelling chronic inflammation, primarily at early stages in the disease, but outlining a treatment for the later stages that lead to plaque rupture. The model is to some extent based on a model of McKay *et al.* [20], although there are significant departures from and elaborations of this model. In particular, features are borrowed from the generally rather simpler models of Cobbold *et al.* [21] and Ibragimov *et al.* [22]. Stability analysis of a simplified version of the model will be performed. We shall be particularly concerned with mechanisms for effects of cholesterol and fractionated low dose radiation exposure in this inflammation model, and outline a case for radiation-induced monocyte cell death as a candidate pathway.

## Models

### Spatial atherosclerosis model

In this section we shall consider a spatial atherosclerosis model based on a simplification of the biology outlined in Text S1 section A.1. The model is entirely concerned with processes in the intima (the tissue immediately adjacent to the endothelial cell lining of the arteries), 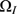, where the disease process is thought to be initiated, with boundary conditions determined in part by species concentrations in the lumen, 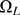. Specifically, the model is concerned with atherosclerosis in the large arteries, for example the coronary, carotid and other cerebral arteries, lesions in which account for the largest part of cardiovascular morbidity and mortality [4]. In Text S1 section A.2 we outline a rather fuller version of this spatial model, incorporating more of the biological detail of section A.1 of Text S1. The main point of the section is the stability analysis that we perform in the final part, this being the reason for the simplifications. The processes are a combination of stage 2 and stage 3 processes outlined in section A.1 of Text S1. The set of reaction-diffusion equations is as follows:
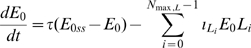
(1)

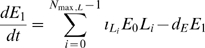
(2)

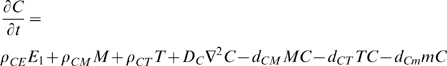
(3)

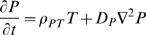
(4)

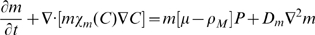
(5)

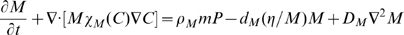
(6)

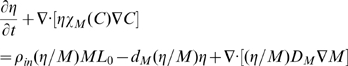
(7)

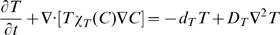
(8)

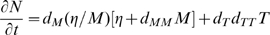
(9)where 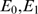 are the undamaged and damaged EC concentrations, 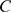 is the chemo-attractant (monocyte chemo-attractant protein 1 (MCP-1)) concentration, 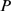 is the proliferation factor (macrophage colony-stimulating factor (M-CSF)), 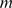 is the monocyte concentration, 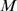 is the macrophage concentration, 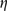 is the bound lipid concentration and 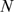 is the necrotic core concentration. 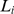 is the LDL concentration with oxidation state 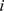 (the number of vitamin E molecules unoxidized - 1) (so 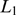 is the LDL with all vitamin E oxidized, although itself unoxidised and 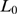 is the fully oxidised LDL concentration). 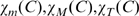 are the chemotactic factors (assumed constant) associated with monocytes, macrophages and T-lymphocytes, respectively; the mechanism for chemotaxis (as given by the terms involving these coefficients) is similar to that of Keller and Segel [23],[24]. 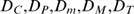 are the rates of diffusion of the associated species. MCP-1 (also known as CCL2) is known to recruit monocytes, T-lymphocytes and dendritic cells to sites of tissue injury. Table S1 gives further details of candidate molecules for some of the model variables.

While many of the components of these equations are standard (further details are given in Text S1 section A), a few deserve further explanation. In equation (3) we assume that chemo-attractant is degraded (via the term 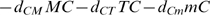) at a rate proportional to the concentration of macrophages, T-cells and monocytes; McKay *et al.* [20] do not assume such degradation. We assume this because chemo-attractant molecules are assumed to adhere to cell-surface markers on these cell species (the mechanism by which they are assumed to attract); a similar assumption was made by Ibragimov *et al.* [22]. In equation (7) we assume that the bound lipid concentration, 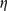, is increased (internalised within macrophages) at a rate determined by the concentration of macrophages, 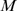, and the concentration of fully oxidized LDL, 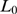 (the 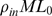 term in equation (7)), but that this bound lipid is released when the macrophages die (the 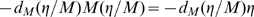 term in equation (7)), a function of macrophage concentration and bound lipid concentration, as given in McKay *et al.* [20]. As discussed in Text S1 section A (equations (A.23)–(A.24) and preceding), we shall assume that 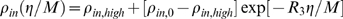 and 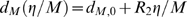. The macrophage flux, as per equation (6), is given by 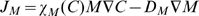. Therefore the bound lipid flux (all carried by macrophage diffusion and chemotaxis) is 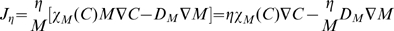. This leads to a chemotaxis term 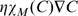 similar to that assumed by McKay *et al.* [20]. We obtain a diffusion term that is entirely due to macrophage diffusion, 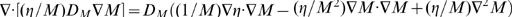, in contrast with McKay *et al.* who assume a standard diffusion term in the lipid, 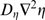; we fail to see how bound lipid can diffuse apart from macrophages - by definition it is bound within macrophages. [
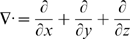
 is the divergence (div) operator, 
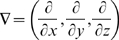
 is the gradient (grad) operator and 
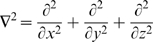
 is the Laplacean operator.] Further details are given in Text S1 section A.

### Boundary conditions

Let 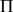 be the outward unit normal on the boundary (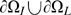), where 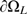 is the boundary between intima and lumen, and 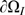 is the boundary between intima and media. Then we have:
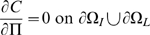
(10)

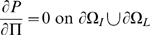
(11)

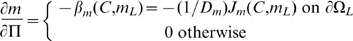
(12)

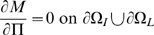
(13)

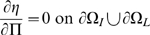
(14)

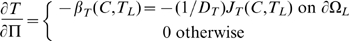
(15)

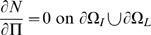
(16)[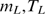 are the monocyte and T-lymphocyte concentrations on the immediate lumenal side of the EC layer.] These boundary conditions are similar to those assumed by Ibragimov *et al.* [22]. Note that in (12) and (15) we convert from the monocyte and T-lymphocyte flux (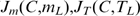 respectively) to the rate of change of concentration per unit distance (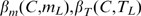 respectively) via the inverse of the respective diffusion constants. We assume the following parametric form of the boundary monocyte and T-lymphocyte flux in (12) and (15):
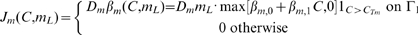
(17)

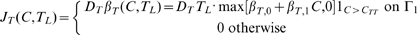
(18)The fundamentally linear form of these is inspired by data in Takaku *et al.* [25] and Klouche *et al.* [26]. The threshold levels of chemo-attractant, 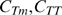, below which these fluxes are zero, is inspired by similar assumptions made by Ibragimov *et al.* [22]; as we discuss below, non-zero threshold levels are needed for there to be a stable solution.

## Results

### Equilibrium solution

**Figure 1:**
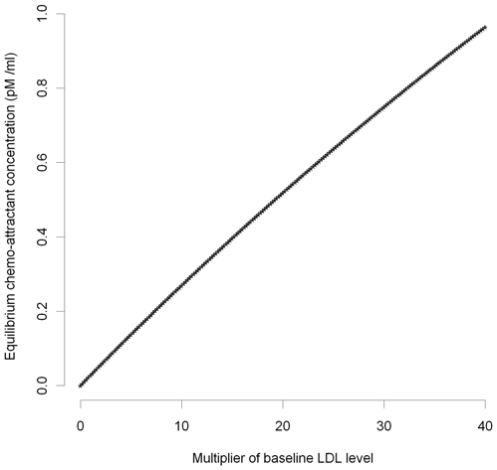
MCP-1 concentration *vs* baseline LDL (small). Equilibrium chemo-attractant (MCP-1) concentration, *C_eq_*, as a function of small multiples of baseline LDL level (Table S3), using parameters given in Tables S2, S3.

**Figure 2:**
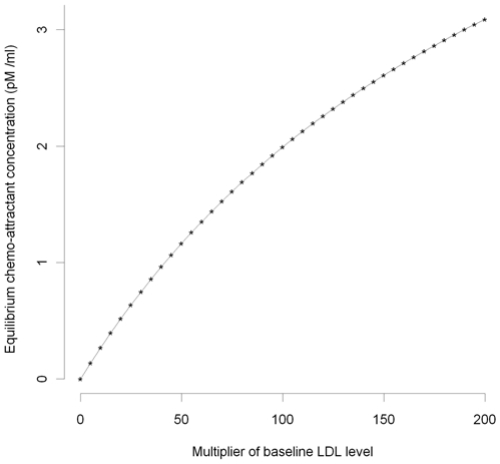
MCP-1 concentration *vs* baseline LDL (large). Equilibrium chemo-attractant (MCP-1) concentration, *C_eq_*, as a function of large multiples of baseline LDL level (Table S3), using parameters given in Tables S2, S3.

We assume that the system is in spatial and temporal equilibrium at some time 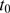, and is subject to some perturbation after that point. Let 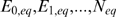 be the equilibrium values of the various quantities, and let 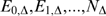 be the differences from these equilibrium values after perturbation – so that, for example, 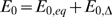, and similarly for the other species. Therefore, for 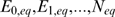 to be the equilibrium values we have by (1)–(9):
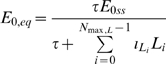
(19)

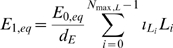
(20)

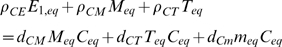
(21)

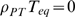
(22)

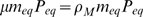
(23)

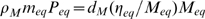
(24)

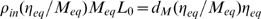
(25)

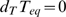
(26)

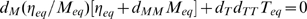
(27)In order that the system be in equilibrium, the boundary monocyte and T-lymphocyte flux must be zero, so we must have that 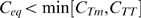; for the remainder of this section we therefore assume this. Assuming the coefficients are non-trivial (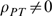, 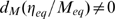, 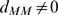, 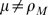) these simplify to (19) and (20) and:
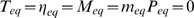
(28)

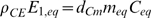
(29)Putting together (19), (20) and (29) we see that:
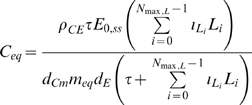
(30)This implies a non-linear relationship between LDL 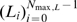 and the equilibrium chemo-attractant level 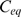. However, as is clear from Figures 1–2, only for very high levels of LDL, multiples in excess of 50 of the baseline levels (see Table S3), are there appreciable departures from linearity.

We shall often assume that 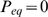. In all that follows we assume a limiting process, so that 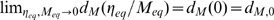, 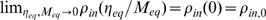. If we perform the obvious linearisations in equations (1)–(9) and ignore all second and higher order terms in 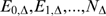 we obtain:
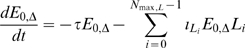
(31)

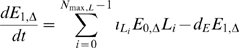
(32)

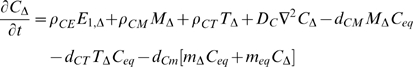
(33)

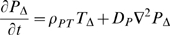
(34)

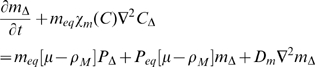
(35)

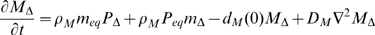
(36)

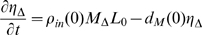
(37)

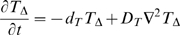
(38)

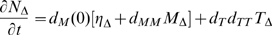
(39)The boundary conditions (10)–(16), together with (17), (18) translate to:
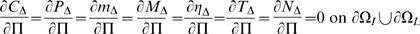
(40)Green's first identity (for general scalar 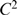 functions 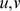 and domain 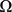) states that:
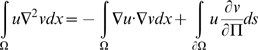
(41)Integrating (33)–(39) over the intima, 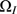, we have by (41) that:
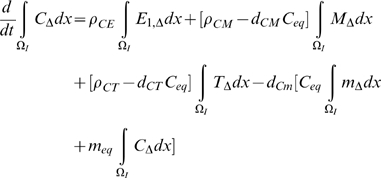
(42)

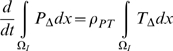
(43)

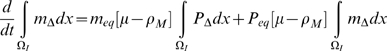
(44)

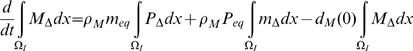
(45)

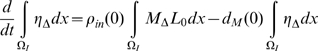
(46)

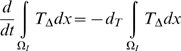
(47)

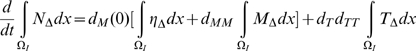
(48)In Text S1 section B we outline solutions to (31)–(32), (42)–(48) in various cases. As shown there, if 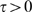 and 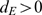 then:
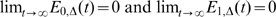
(49)From Table S2 it is clear that these conditions are likely to be always satisfied: 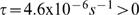 and 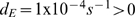. If 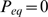 then using the results of Text S1 section B ((B.11)–(B.16)):
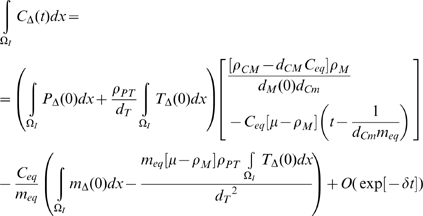
(50)

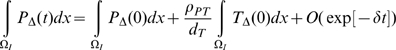
(51)

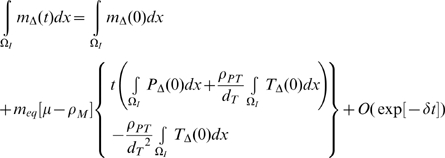
(52)

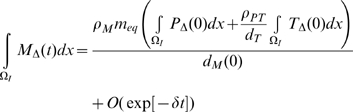
(53)

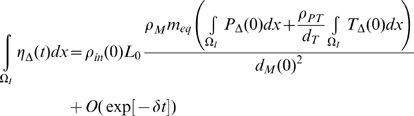
(54)

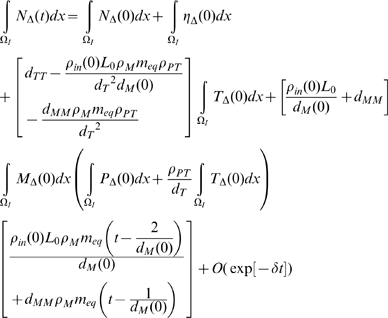
(55)for some 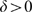, so that in general 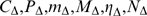 do not decay to 0 as 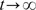, although by (B.18) 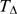 does (at least in 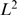). Note in particular that unless 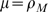 and 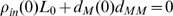, or possibly (more likely) that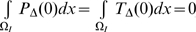, then there will be time trends in the averaged quantities for 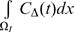, 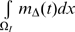 and 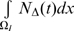, so that in particular stability cannot be re-attained.

**Figure 3:**
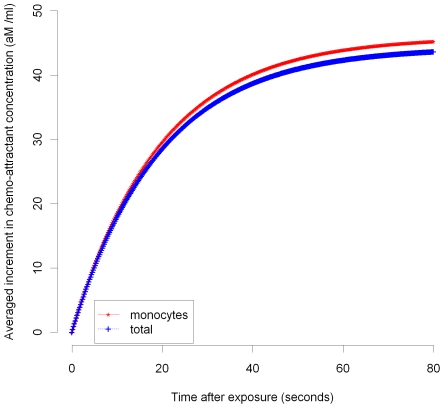
MCP-1 variation over 0–80 seconds after 10 mGy (monocyte, total). Spatial average (over intima) of increment in chemo-attractant (MCP-1) concentration after 10 mGy of acutely delivered radiation, using parameters given in Tables S2, S3. The components of changes in chemo-attractant (MCP-1) level due to monocyte cell killing and total (monocyte+endothelial) cell killing 0–80 seconds after 10 mGy are shown.

In Figures 3–7 we plot the variation of the spatially-averaged chemo-attractant, 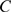, derived assuming a non-zero equilibrium concentration of monocytes (Table S3) and using (B.1b’). The perturbation is assumed to take place via killing of monocytes in the intima, which in this case could be produced by ionizing radiation, and also via damage to endothelial cells, produced in the same way. We do not assume instantaneous changes in any of the other species, i.e., 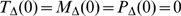. The reason for this is that by (28) 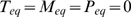 (assuming as we do that 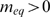), so that radiation would not have any species to act on in equilibrium. For the parameters used here (given in Tables S2 and S3), the overwhelming contribution is via monocyte killing: by 80 seconds the contribution from this term is 4.5×10^−17^ M ml^−1^ compared with a contribution of −1.6×10^−18^ M ml^−1^ via damage to endothelial cells. As can be seen, the change in chemo-attractant concentration occurs (for monocytes and in aggregate) relatively quickly, over a timescale of minutes, although the endothelial cell killing component varies more slowly, over a timescale of hours; after 24 hours this and all other averaged quantities are virtually constant. In contrast to the above general case, when only the monocyte population (of all the species) is perturbed, for a sufficiently long time after exposure (days or more) we have by (50), (52) that 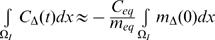 and 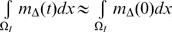, and by (51), (53), (54) the averaged change in all other species tends to zero. In this case we see that:
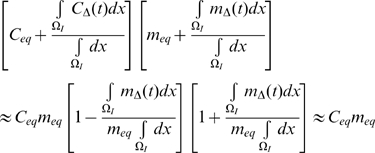
(56)approximated to first order, so that by (30), at least in average, equilibrium can be re-established at these new values of 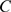 and 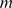. We conjecture that in fact equilibrium is re-established for all quantities in this case. It is easy to see that if there were to be further small perturbations in 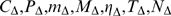, at intervals of days or more, the resulting changes in the spatially-averaged quantities would be approximately additive in the corresponding increments, as shown in Figure 7. Moreover, from (50) the excess chemo-attractant (MCP-1) in relation to the monocyte perturbation is 
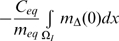
. Therefore, so long as the individual monocyte perturbations are small and temporally separated (by a day or more), the increment in chemo-attractant will not depend on anything other than the cumulative absorbed dose, as indicated in Figure 7.

**Figure 4 pcbi-1000539-g004:**
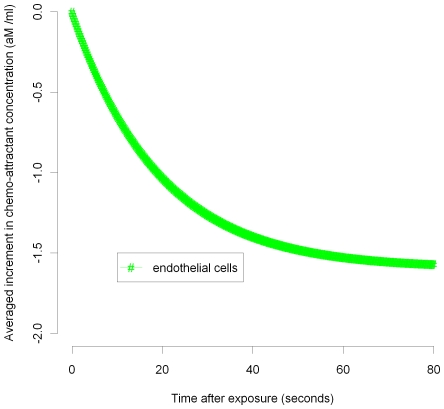
MCP-1 variation over 0–80 seconds after 10 mGy (endothelial). Spatial average (over intima) of increment in chemo-attractant (MCP-1) concentration after 10 mGy of acutely delivered radiation, using parameters given in Tables S2, S3. The component of changes in chemo-attractant (MCP-1) level due to endothelial cell killing 0–80 seconds after 10 mGy are shown.

**Figure 5 pcbi-1000539-g005:**
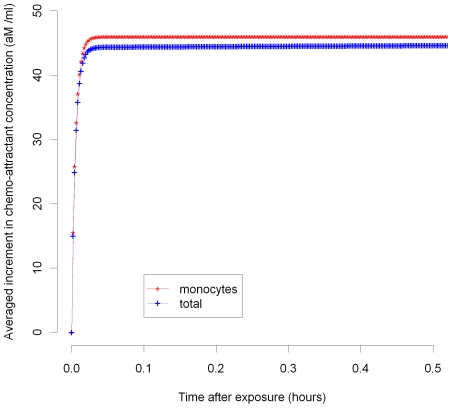
MCP-1 variation over 0–0.5 hours after 10 mGy (monocyte, total). Spatial average (over intima) of increment in chemo-attractant (MCP-1) concentration after 10 mGy of acutely delivered radiation, using parameters given in Tables S2, S3. The components of changes in chemo-attractant (MCP-1) level due to monocyte cell killing and total (monocyte+endothelial) cell killing 0–0.5 hours after 10 mGy are shown.

**Figure 6 pcbi-1000539-g006:**
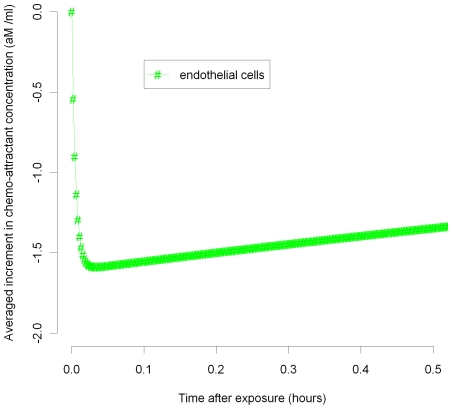
MCP-1 variation over 0–0.5 hours after 10 mGy (endothelial). Spatial average (over intima) of increment in chemo-attractant (MCP-1) concentration after 10 mGy of acutely delivered radiation, using parameters given in Tables S2, S3. The component of changes in chemo-attractant (MCP-1) level due to endothelial cell killing 0–0.5 hours after 10 mGy are shown.

**Figure 7:**
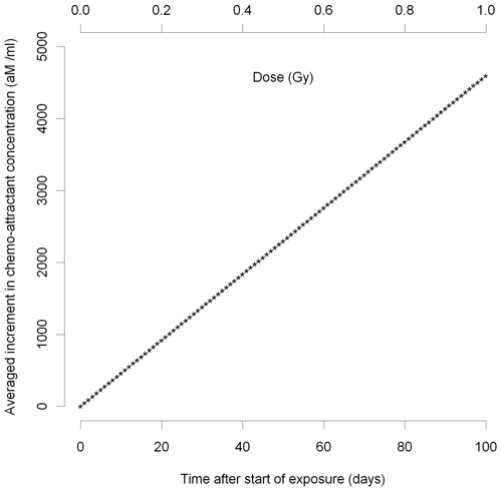
MCP-1 variation over time after 10 mGy/day. As for Figures 3–6, but assuming fractionated multiple radiation doses, 10 mGy/day.

**Figure 8:**
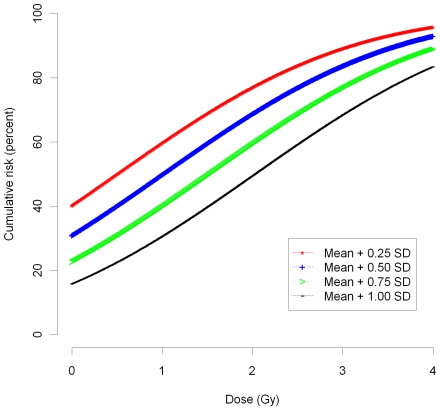
Risk of MCP-1 exceeding threshold level *vs* dose. Cumulative risk of exceeding of chemo-attractant (MCP-1) threshold (≈cumulative risk of cardiovascular disease) min[*C_Tm_*,C*_TT_*], as function of radiation dose and threshold value (mean+multiple of population SD [27] (see Table S3)).

In Figure 8 we plot the percent proportion of the population whose cumulative chemo-attractant (MCP-1) concentration exceeds the threshold 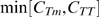; as we discuss below, this threshold is the critical point for system stability, exceedance of which makes development of cardiovascular disease much more likely. [The probability is derived assuming that the population distribution MCP-1 is Gaussian with mean and standard deviation (SD) determined by the adult female data of Cannon *et al.* [27]; the mean is augmented by the radiation-induced increment, given by (B.1b’).] For a range of threshold values between 0.25 and 1.00 times the SD in excess of the mean, we have baseline risks of exceeding the threshold (i.e., cardiovascular disease) of 16–40%. Most developed countries have cumulative cardiovascular disease mortality in the range 20–40% and the world mean is 30% [28], so that this range of values of the MCP-1 threshold, 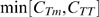, is plausible. For this range of threshold values, Figure 8 demonstrates that risks vary remarkably linearly with dose over the dose interval 0–4 Gy. As for Figure 7, the risk will not depend on anything other than the cumulative absorbed dose, as long as this is given in small daily increments.

## Discussion

We have outlined a model for early stage atherosclerotic lesion formation, and performed a stability analysis for a simplified version of the model. While some components of the system (in particular the T-lymphocyte concentration, 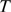) are stable, in the sense that after perturbations of the system the species concentrations return to their equilibrium value, various other species, in particular the proliferation factor concentration, 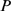, the chemo-attractant concentration, 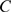, the monocyte concentration, 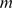, and the necrotic core, 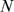, are generally not stable. In particular, the mean level of chemo-attractant increases continuously and rapidly after instantaneous perturbation by a radiation dose, over a timescale of minutes. However, as we note below, because of cellular repair processes, which are not taken into account in our model, there are reasons for assuming that perturbation by radiation would not be instantaneous, so that this process might be extended over at least a period of hours after exposure. The main driver for the increase in chemo-attractant is the death of monocytes and the consequent reduction in monocyte-induced degradation in chemo-attractant concentration, the 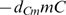 term in (3). It is well known that radiation can cause cell death [29], and the degree of cell killing and damage that we assume is consistent with radio-biological expectation [30],[31]. Although the change in chemo-attractant (MCP-1) concentration that we assume after 10 mGy is relatively modest, 4.5×10^−17^ M ml^−1^, a fractionated dose of 1 Gy would result in 4.5×10^−15^ M ml^−1^, comparable with the normal concentration of MCP-1 in adult plasma, 7.9×10^−15^ M ml^−1^ [27]. The fact that the range of excess relative risks predicted by our model, 0.49–0.93 Gy^−1^, is consistent with those in a number of occupational studies (Table 1) adds to the plausibility of this mechanism.

**Table 1:** Risks in various human cohorts, and predicted by model.

Data	Reference	Endpoint (mortality unless otherwise indicated)	Excess relative risk at 1 Gy (and 95% CI)
Japanese atomic bomb survivors	Preston *et al.* [8]	Heart disease, 1968–1997 (ICD9 390–429)	0.17 (0.08, 0.26)^a^ ^b^
		Stroke, 1968–1997 (ICD9 430–438)	0.12 (0.02, 0.22)^a^ ^b^
Mayak workers	Azizova and Muirhead [14]	Ischaemic heart disease morbidity (ICD9 410–414)	0.109 (0.049, 0.168)
		Cerebrovascular disease morbidity (ICD9 430–438)	0.464 (0.360, 0.567)
Chernobyl emergency workers	Ivanov *et al.* [11]	Cerebrovascular disease (ICD10 I60–I69)	0.45 (0.11, 0.80)
		All circulatory disease (ICD10 I00–I99)	0.18 (−0.03, 0.39)
German uranium miner study	Kreuzer *et al.* [56]	All circulatory disease (ICD10 I00–I99)	−0.26 (−0.6, 0.05)
		Heart disease (ICD10 I00–I52)	−0.35 (−0.7, 0.009)
		Cerebrovascular disease (ICD10 I60–I69)	0.09 (−0.6, 0.8)
BNFL workers	McGeoghegan *et al.* [12]	Ischaemic heart disease (ICD9 410–414)	0.70 (0.37, 1.07)^a^ ^b^
		Cerebrovascular disease (ICD9 430–438)	0.66 (0.17, 1.27)^a^ ^b^
		All circulatory disease (ICD9 390–459)	0.54 (0.30, 0.82)^a^ ^b^
UK National Registry for Radiation Workers	Muirhead *et al.* [13]	All circulatory disease (ICD9 390–459)	0.251 (−0.01, 0.54)^b^
US Oak Ridge workers	Richardson and Wing [57]	Ischaemic heart disease (ICD8 410–414)	−2.86 (−6.90, 1.18)^b^ ^c^
IARC 15- country nuclear worker study	Vrijheid *et al.* [15]	Circulatory disease (ICD10 I00–I99, J60–J69, O88.2, R00–R02, R57)	0.09 (−0.43, 0.70)^b^
		Cerebrovascular disease (ICD10 O88.2)	0.88 (−0.67, 3.16)^b^
Predicted by model, based on chemo-attractant (MCP-1) concentration			0.58^d^
			0.49–0.93^e^

Excess relative risks (per Gy) of cardiovascular disease in the Japanese atomic bomb survivors and in various occupationally exposed groups, compared with excess relative chemo-attractant (MCP-1) concentration at 1 Gy predicted by model.

a 90% CI.

b Excess relative risk Sv^−1^.

c Assuming 10 year lag.

d 1 Gy assumed given as 100 daily doses of 10 mGy, ERR evaluated by dividing excess MCP-1 concentration by baseline level from data of Cannon *et al.* [27].

e ERR at 1 Gy of cumulative risk of exceeding threshold (≈cumulative cardiovascular risk), as given by Figure 8, for levels of MCP-1 threshold min[*C_Tm_*,C*_TT_*] in the range [mean+0.25 SD, mean+1.00 SD], mean and SD taken from data of Cannon *et al.* [27].

We have also shown that the model predicts that equilibrium level of chemo-attractant (MCP-1) increases more or less directly with levels of LDL, and in particular oxidized LDL, with slight non-linearity at very high levels of MCP-1. This is in accordance with experimental [32],[33] and epidemiological observations [34]. Specifically, there is experimental evidence that addition of minimally-oxidised LDL results in a ≈22-fold increase in levels of MCP-1 in ECs in an *in vitro* co-culture system [32]. In a group of baboons fed a high cholesterol, high fat diet, oxLDL in serum increased by about 19.6% (95% −28.9, 68.1) after 7 weeks, resulting in an increase in serum MCP-1 at that point of 66.7% (95% 54.2, 79.1) [33]. Both of these are consistent with the linear relationship (without constant term) predicted by our model (30).

If radiation dose were to be given in a fractionated manner, with doses separated by a period of hours or more, the model predicts that chemo-attractant (e.g., MCP-1) would increase linearly with cumulative accumulated dose, with a corresponding decrease in the intimal monocyte concentration, as shown in Figure 7. This would carry on until the chemo-attractant concentration at the boundary, 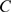, exceeds one or other of the thresholds 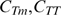, beyond which point an equilibrium solution is no longer possible. At these points, there would be increased trans-intimal flux of monocytes and T-lymphocytes from the lumen, which would result (via (48)) in a continuous increase in necrotic lesion size, and therefore risk of atherosclerosis. The doses used here are moderate (10 mGy/day), such as might occur in occupational exposure settings, and would account for the observed radiation-associated excess risk that has been seen in various groups of nuclear workers [10]–[14]. The model implies that at least until the chemo-attractant threshold 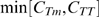 is exceeded the system is stable, assuming that the conjecture we make after (56) is valid.

If the chemo-attractant threshold 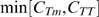 is exceeded as a result of the perturbation term, 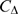, resulting in monocyte or T-lymphocyte flux across the EC layer, then extra terms need adding to the right hand side of (44) and (47),
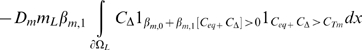
(57)and
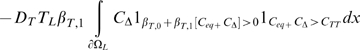
(58)respectively. Apparently paradoxically, if 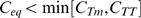, then we must have 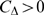 for the terms inside the integrals to contribute non-trivially, and so these terms will be negative and therefore tend to reduce the averaged levels of monocytes and T-lymphocytes in the system. By (42) this will tend to increase the chemo-attractant concentration still further. In other words, once this chemo-attractant threshold is crossed the system tends (on average) to become yet more unstable.

There are of course other agents that damage monocytes or ECs that would cause the chemo-attractant level to increase, so that although for an individual this threshold might never be passed, in a large population there would be a continuous (and approximately linear) increase in cardiovascular risk with dose as shown in Figure 8. The same phenomenon would also occur at higher doses (e.g., at radiotherapeutic levels of dose), at a correspondingly higher level, although the relative magnitude of the perturbations would make the neglect of all but first order perturbations that we assumed in deriving (31)–(39) possibly invalid; there is abundant evidence of radiation-induced disease in groups exposed to certain forms of RT [6],[7]. Critical to our model, and indeed the understanding of atherosclerosis, is whether there really are such thresholds in chemo-attractant levels for the trans-intimal monocyte and T-lymphocyte flux. We assume the presence of such thresholds for the purposes of our stability analysis, as we have to if there is to be a stable solution, but it is possible nevertheless that these thresholds are zero, in which case, assuming the model is correct, the atherosclerotic process must be inherently unstable. As indicated above, if this is model is correct and is to be consistent with the observed cumulative cardiovascular disease mortality in developed populations [28], then the chemo-attractant (MCP-1) threshold must lie in the range [mean+0.25×population SD, mean+1.00× population SD] (the mean and population SD being as in Cannon *et al.* [27]), in other words [1.0, 1.7]×10^−14^ M ml^−1^.

We implicitly assume that atherosclerosis is mainly responsible for the observed excess risk of cardiovascular morbidity or mortality following fractionated low-dose irradiation of the heart and major arteries. This assumption is supported by experimental data in *ApoE* ^−/−^ mice [35]. However, some human symptoms are due to (myocardial) ischaemia which could be caused by either macrovascular (atherosclerotic) or microvascular damage. At higher (radiotherapy) doses, both human and animal data suggest that both types of lesion occur [6]. Although the generally high prevalence of atherosclerosis in humans suggests that this is the more probable cause of ischaemia following low-dose radiation, it is possible that microvascular disease also plays a role. It should be noted that we have been addressing mechanisms for induction of atherosclerosis following fractionated low-dose radiation to the large arteries (coronary, carotid etc). There is a large literature on fibrotic, pericardial, myocardial and other morbidity sequelae of high-dose irradiation of the heart and large arteries, both for humans and animals [6]. The pro-inflammatory mechanisms for these are reasonably well understood, and quite different from those hypothesized here [36]. That the true mechanisms for low-dose effects are likely to be very different is also suggested by the pronounced fractionation effect seen for high-dose exposure in relation to heart failure in rats [37],[38], in contrast to the somewhat lower risks observed in the Japanese atomic bomb survivors compared with occupationally exposed groups (Table 1).

Indirect mechanisms for the action of radiation could also be postulated. At high doses it is clear that inflammatory markers are up-regulated *in vitro* and *in vivo*, although at lower doses if anything the evidence points to down-regulation of inflammation [16]. In terms of the model this could be mediated by an increase in radical flux, which could, via lipid peroxidation, lead to EC damage. This in turn would lead to an increase in the chemo-attractant signal. Radiation is known to cause long-term variation in certain T-cell subpopulations (CD4+) in the Japanese atomic bomb survivors [39], and this mechanism could also be readily incorporated in the model. Long-term radiation-associated changes in cholesterol concentration have been observed in the Japanese atomic bomb survivors [40], presumably a result of some change in liver metabolism; these too could be easily incorporated in the model. It is of interest in this respect that there is a highly statistically significant trend with internal (plutonium α-particle) dose to the liver for ischaemic heart disease and cerebrovascular disease in the latest analysis of the Mayak worker data [14]. Set against that, there is little evidence of excess risk of circulatory disease risk, specifically cardiac disease in groups exposed to the diagnostic contrast medium Thorotrast, which delivered a substantial α-particle liver dose [41],[42].

An important consideration in estimating dose to the intima, and which may have a bearing on interpretation of certain epidemiological studies, is the role of oxygen diffusion. This has been modelled by Richardson [43]–[45], who has highlighted the pronounced variations with oxygen concentration across the intima, which also varies with age as a result of modifications in arterial geometry [44]. It is well known that with decreasing oxygenation the effective dose reduces [45], and this implies that biologically effective dose per unit exposure reduces by 8–12% from age 0.5 to 70 years, whether for high linear energy transfer (LET) (^222^Rn, ^218^Po, ^214^Po) or for low LET radiation [45]. This needs to be addressed in the dosimetry of any study; assuming that, as we argue above, intimal dose is of the most relevance to cardiovascular risk, not doing so would imply a modest negative bias in modifications of the radiation response by age at exposure.

Whilst the inflammatory process is recognized as an integral part of the atherosclerotic process [5] it does not explain the observation that the proliferation of vascular smooth muscle cells (VSMC) during atherosclerotic plaque development appears to be monoclonal [46]. Clonality suggests that plaque VSMCs must have undergone multiple rounds of division, and telomere loss studies argue that this is between 7–13 cumulative population doublings [47]. However, clonality itself is not synonymous with transformation of a single cell, and subsequent studies have shown that large patches in the normal vessel media are monoclonal [48],[49]. Thus, clonality is more likely to be explained by the presence of developmental clones in the normal vessel wall, rather than a mutation. Finally, in contrast to tumours, plaque VSMCs show poor proliferation, enhanced apoptosis, and early senescence [50]. These features would not confer a proliferative or survival advantage to plaque VSMCs. Furthermore, plaque VSMC proliferation is now seen to be beneficial in atherosclerosis [51], so that the pathological consequences of a mutation promoting VSMC proliferation are unclear.

The limitations of the modelling performed here should be acknowledged. Even in the fuller model considered in Text S1 section A there is much biology not included – simplifications have been made for analytical simplicity. Although not strictly a defect in the model, we assume in our motivating example that a certain (dose-dependent) fraction of the monocytes are killed instantaneously by radiation exposure. The magnitude of this fraction is based on data from a human bone-marrow colony-forming assay (for cells under hypoxic conditions) of Gordon [30] (Table S3), performed 9 days after irradiation. It is known that cells take a variable length of time to die after irradiation, as a result of the repair and mis-repair processes they are thought to be subject to [52]. As such, a possibly more realistic scenario would have assumed this total cell damage exponentially distributed over time rather than occurring instantaneously. However, it is unlikely that the variable delay in expression of monocyte mortality, which is likely to be 99.7% complete within three hours of irradiation [52], will make much difference to the predictions of our model, concerned as it largely is with the consequences of fractionated radiation doses separated by days or more. It would not be too difficult to modify the equations (5), (6) and (8) to incorporate the simple repair-misrepair model outlined in Brenner *et al.* [52], although for the purposes of the present paper we regard this as an unnecessary elaboration.

That said, the simpler model proposed here we trust captures what is known about the main features of interaction of oxidized LDL and various other molecular species (MCP-1, G-CSF, bound lipid) with the various cellular species (monocytes, macrophages, T-lymphocytes) that are known to be of significance for induction of atherosclerosis. The mathematics underlying these reaction and diffusion processes is reasonably standard. What is interesting and novel about the present paper is that using only experimentally derived parameters (taken wherever possible from human data) (Tables S2, S3) we have reproduced what is observed in other experimental and epidemiologic data (Figures 7–8, Table 1).

This proposed mechanism would in principle be experimentally testable. This would best be done *in vitro*, looking for changes in MCP-1 levels, or other potential chemo-attractants, in a co-culture system similar to that developed by Takaku *et al.* [25]. This could be explored under a range of radiation exposure conditions (both localized and fractionated) and subsequent effects on, for example, adhesion properties could also be examined. *In vivo* experiments would be more complex (and expensive), but could also be performed, for example, using the *ApoE* ^−/−^ knockout mouse model employed by Stewart *et al.* [35],[53]. Even human data could be envisaged. In particular, if arterial tissue could be sampled from patients who have, a short time previously, received low-dose radiotherapy or high-dose diagnostic procedures (e.g., computerized tomography), together with suitable (age-matched) controls, one could determine whether intimal concentration of MCP-1 was significantly increased and the manner in which concentration changed with dose.

If the proposed mechanism were true, it also has substantive implications for radiological protection, which at present does not take cardiovascular disease into account [54]. Analysis of the Japanese atomic bomb survivor data implies that non-cancer disease mortality, in particular cardiovascular mortality, contributes almost equally as cancer mortality to the radiogenic excess risk [8]. The major uncertainty in assessing the low-dose risk of cardiovascular disease is the shape of the dose response relationship, which is very unclear in the Japanese data [8],[55]. The analysis of the present paper suggests that linear extrapolation would be generally appropriate for this endpoint.

## Supporting Information

Text S1 Text S1 (sections A, B)
(0.46 MB DOC)

Table S1 Candidate molecules for variables in the model.
(0.04 MB DOC)

Table S2 Parameters and estimated values.
(0.35 MB DOC)

Table S3 Values for perturbed species.
(0.07 MB DOC)
