# Supplementary material for: A Model of Cardiovascular Disease Giving a Plausible Mechanism for the Effect of Fractionated Low-Dose Ionizing Radiation Exposure
Source: PLoS Comput Biol. 2009 Oct 23;5(10):e1000539. doi: 10.1371/journal.pcbi.1000539 (PMC2759077; doi:10.1371/journal.pcbi.1000539)
Supplement: Text S1 — (sections A, B) (0.46 MB DOC) [file pcbi.1000539.s001.doc]

**Supplementary material A.**

**The biology of atherosclerosis and a more complete reaction-diffusion model**

In this section we shall present an outline of the biology of atherosclerosis (section A.1). We go on to outline a mathematical model based on these ideas (section A.2). It is a rather fuller version of the reaction-diffusion model treated in the main text. This fuller model is close to that of McKay *et al.* [1], although some processes, in particular diffusion and chemotaxis are different. In this Appendix we shall also present (Table S1) candidate molecules for some of the modelled quantities. Table S2 contains a list of all model parameters and values where known, generally derived from *in vitro* and *in vivo* experimental systems, and wherever possible based on human material.

**A.1 Review of Biological Processes**

**A.1.1 Stage 1: Lipoproteins enter the intima and undergo oxidation**

In atherosclerosis, the normal functions of the endothelium are altered, instigating an inflammatory process [2]. Through mechanisms such as shear stress [3], a portion of the endothelial layer of a muscular arterial wall develops a “leaky” spot permitting accelerated transport of LDL (and other macromolecular species) through the endothelial barrier into the intima, where they tend to concentrate due to the difficulty of further passage through the inner elastic lamina into the media [4], and where they become oxidized (oxLDL), via vascular oxidative waste products.

Among the most critical reactions are the oxidation and anti-oxidation reactions involving LDL and HDL. LDL is chemically modified by reactive oxygen species (ROS) produced through metabolic processes occurring in cells within the arterial wall (e.g., vascular smooth muscle cells (VSMC), endothelial cells (EC), fibroblasts). When a ROS comes into contact with a lipoprotein, initially it reacts with vitamin E, its natural defence. To be fully oxidized, all vitamin E defences (on average 6 [5, 6]) have to be depleted. However, as yet there is no evidence from clinical trials to suggest that natural anti-oxidants (beta-carotene, vitamin C) can reduce damage caused by oxidized LDL [7]. Oxidised low density lipoprotein (LDL) is one of the factors contributing to the initiation of the atherosclerotic process [8], as we outline below.

In addition to LDL, high density lipoprotein (HDL) particles are also present in the blood, whose role is primarily the reverse transport of lipid back to the liver. HDL is capable of inhibiting oxidative modification of LDL due to its anti-inflammatory and anti-oxidant properties. However, some studies have shown that HDL can also elicit a pro-inflammatory response in macrophages and hence does not provide protection to LDL against oxidation [9].

**A.1.2 Stage 2: The inﬂammatory process**

Monocytes and T-lymphocytes enter the intima in response to molecular signalling from an initiating inflammatory reaction (e.g., due to viral or bacterial insult, oxLDL) [10]. In particular when injured (e.g., by oxLDL) ECs emit adhesion molecules (on the lumenal side), growth factors (e.g., macrophage colony stimulating factor (M-CSF)) and chemo-attractants (e.g., monocyte chemo-attractant protein 1 (MCP-1)). Other inflammatory cells (e.g., monocyte-derived macrophages) also accumulate in arteries. Macrophages seek out and engulf apoptotic or foreign bodies. Macrophages have an affinity for the oxLDL – indeed, there is strong experimental evidence (e.g., ref. [11]) that macrophages exhibit positive chemotactic sensitivity to these oxLDL species. Macrophages release growth factors/cytokines (e.g., platelet-derived growth factor (PDGF), transforming growth factor-beta (TGF-*β*), granulocyte-M-CSF). These cytokines enhance LDL-receptor activity, perhaps due in part to the reduction of intracellular free cholesterol [12]. Due to the enhanced LDL-receptor activity, an increased amount of cholesterol is delivered to the cell.

Eventually, the cell wall becomes lipid-engorged, as it is unable to traffic the large amounts of LDL-cholesterol esters, and the macrophage becomes a foam cell. It is then incapable of doing its customary job of removing the debris produced by the inflammatory processes. In addition, lipoprotein entrapment by the extracellular matrix can lead to the progressive oxidation of LDL, because of the action of ROS. A range of oxLDL is thus generated, ultimately resulting in its delivery to vascular cells through various families of scavenger receptors [12]. These cellular “saboteurs”, once formed or deposited in the cell, can contribute to and participate in the formation and accumulation of cholesterol esters and subsequent transformation of a macrophage into a foam cell.

**A.1.3 Stage 3: Plaque growth: smooth muscle cell migration and apop­tosis in the vasculature**

A lipid-enriched fatty streak along the arterial wall can develop. Because antioxidant defences may be limited in the microenvironment of the cell or within LDL, the oxidation process continues to progress. The growing lesion produces various molecular signalling species that attract additional macrophages to the lesion site which then get “corrupted” by the oxLDL species, resulting in a chronic inflammatory spiral [2, 13]. Another aspect of plaque growth involves the recruitment (via molecular signalling) of VSMCs to the lesion site and their role in forming a tough cap to isolate the lesion from healthy tissue and the lumen. However, in contrast to the classic paradigm, it is now clear that VSMC proliferation actually limits the growth of the atherosclerotic lesion [14]. Related to this, VSMC apoptosis is known to accelerate atherosclerotic lesion formation [15]. VSMCs also secrete chemo-attractants, in particular MCP-1 [16], into the intima. VSMCs reside in the layer beneath the intima (the media), segregated by a band of elastic tissue. Most advanced atherosclerotic lesions consist of a lipid core surrounded by a fibrous cap of VSMCs and connective tissue. In the media, VSMCs are in a contractile, non-mobile phase. However, these cells may be chemically stimulated to become mobile and migrate into the intima to surround a forming lesion [17]. As the lesion forms, the arterial wall may undergo remodelling (expansion). Continued growth results in lumenal encroachment.

There is also evidence to suggest that VSMCs respond to mechanical stress [18]. Therefore it may be that growth of atherosclerotic plaque due to cell apoptosis alters the mechanical stress (stretch) felt by the medial (VSMC) layer and induces VSMC migration from the media to the intima [19].

**A.1.4 Stage 4: Plaque instability: vascular smooth muscle cell and endothelial cell apoptosis and cap degradation**

Research suggests that the primary cause of acute coronary events is break-down of the ﬁbrous cap and the consequent formation of a thrombus within the lumen. Indeed, only 15% of heart attacks and strokes are caused by the complete occlusion of the lumen by plaque growth [20].

The growth of atherosclerotic plaque does not, initially, grow into the lumen. The majority of plaque growth is into the medial (VSMC) layer and it is only after this growth has reached some limit (the artery is no longer able to compensate by dilation) that it encroaches on the lumen. The presence of such stenosis is disruptive to the local blood ﬂow and consequently the shear stress across the occlusion can vary. This variation in shear stress is thought to be the cause of plaque cap rupture. The rupturing of the atherosclerotic plaque cap can be linked with a large number of diﬀerent factors. However, crucially, all these factors lead to the destabilising and eventual rupturing of the plaque cap through cell apoptosis.

VSMC apoptosis and senescence is harmful as it weakens the ﬁbrous cap by decreasing the synthesis of extracellular matrix [14, 15] and therefore may lead to plaque rupture. It is well known that balloon angioplasty induces rapid death of VSMCs within the normal interior wall showing that mechanical stress is an important factor in VSMC apoptosis [19]. Ox-LDL has also been shown to promote VSMC apoptosis. EC apoptosis is also an important factor in plaque rupture. Recently it has been shown that EC apoptosis is signiﬁcantly increased in the downstream area of the plaque, where shear stress is low and blood ﬂow disturbed [21]; this agrees with evidence that ruptures are primarily found on the shoulder of plaques. However, apoptosis inside the plaque (that is apoptosis of macrophages/VSMCs etc) is found to be higher in areas associated with high stretch stress upstream of the plaque [19]. In addition to VSMC and EC apop­tosis, matrix metalloproteinase (MMP) secreted by macrophages [8] can actively destroy the extracellular matrix and contribute to instability in an atherosclerotic plaque.

**A.2 Model of atherosclerosis**

In this section we shall consider a model based on the biological ideas outlined above. The model is constructed in a number of stages, corresponding to the various biological stages outlined in section A.1.

**A.2.1 Stage 1 processes (occurring in both the lumen and the intima)**

For modelling purposes we will assume (as did McKay *et al.* [1]) that monocytes and T-cells may be adequately described by their function in the stage 2 processes in the intima only. Therefore we have the following reaction-diffusion partial differential equations describing the stage 1 processes in the intima:

$\frac{\partial L_{i}}{\partial t}=k_{L_{i+1}}rL_{i+1}-k_{L_{i}}rL_{i}-\omega_{L_{i}}vL_{i}+\omega_{L_{i-1}}vL_{i-1}+D_{L_{i}}\nabla^{2}L_{i} (1\leq i\leq N_{\max,L})$ (A.1)

$\frac{\partial L_{0}}{\partial t}=-\rho_{in}ML_{0}+k_{L_{1}}rL_{1}-\omega_{L_{0}}vL_{0}+D_{L_{0}}\nabla^{2}L_{0}$ (A.2)

$\frac{dH_{i}}{dt}=k_{H_{i+1}}rH_{i+1}-k_{H_{i}}rH_{i}-\omega_{H_{i}}vH_{i}+\omega_{H_{i-1}}vH_{i-1}+D_{H_{i}}\nabla^{2}H_{i} (1\leq i\leq N_{\max,H})$ (A.3)

$\frac{dH_{0}}{dt}=k_{H_{1}}rH_{1}-\omega_{H_{0}}vH_{0}+D_{H_{0}}\nabla^{2}H_{0}$ (A.4)

$\frac{\partial r}{\partial t}=-rd{}_{ox} \left(\sum_{i=1}^{N_{\max,L}} k_{L_{i}}L_{i}+\sum_{i=1}^{N_{\max,H}} k_{H_{i}}H_{i}\right)+D_{r}\nabla^{2}r$ (A.5)

$\frac{dv}{dt}=-vd_{antox}\left(\sum_{i=0}^{N_{\max,L}-1} \omega_{L_{i}}L_{i}+\sum_{i=0}^{N_{\max,H}-1} \omega_{H_{i}}H_{i}\right)+D_{v}\nabla^{2}v$ (A.6)

where $H_{i}$ is the HDL concentration with oxidation state $i$ (the number of vitamin E molecules unoxidized - 1) (so $H_{1}$ is the HDL with all vitamin E oxidized, although itself unoxidised, $H_{0}$ is the oxidised HDL concentration), $r$ is the radical concentration, $v$ is the anti-oxidant concentration, $k_{L_{i}},k_{H_{i}}$ are the rates (per unit radical concentration) of oxidation of LDL and HDL (with oxidation state $i$) and $\omega_{L_{i}},\omega_{H_{i}}$ are the rates (per unit anti-oxidant concentration) of de-oxidation of LDL and HDL (with oxidation state $i$). $d_{ox}$ is the rate of reduction of free radical as a result of oxidation reactions with vitamin E and HDL and $d_{antox}$ is the rate of reduction of anti-oxidant as a result of anti-oxidation reactions with vitamin E and LDL or HDL; it is assumed that this is proportional to the reaction rates with the respective LDL and HDL species, as also did McKay *et al.* [1] and Cobbold *et al.* [22]. $\rho_{in}$ is the rate of conversion per unit of fully oxidized LDL and per unit macrophage concentration to lipid-bound LDL (i.e. to LDL ingested into macrophages). All other species are as in section 2 of the main text. It should be noted that we explicitly model the various oxidation stages of LDL and HDL, which McKay *et al.* [1] do not. This model was suggested by Cobbold *et al.* [22]. The oxidation states of HDL are also explicitly modelled, a feature of the present model that was not taken into account by McKay *et al.* [1], although it was by Cobbold *et al.* [22]. $D_{L_{i}},D_{H_{i}},D_{r},D_{v}$ are the rates of diffusion of LDL, HDL (oxidation state $i$), radicals and anti-oxidants, respectively. In the lumen, a similar set of equations holds for the stage 1 processes, but account must also be taken of the blood-flow velocity $u$:

$\frac{\partial L_{i}}{\partial t}+u\cdot\nabla L_{i}=k_{L_{i+1}}rL_{i+1}-k_{L_{i}}rL_{i}-\omega_{L_{i}}vL_{i}+\omega_{L_{i-1}}vL_{i-1}+D_{l,L_{i}}\nabla^{2}L_{i} (1\leq i\leq N_{\max,L})$ (A.7)

$\frac{\partial L_{0}}{\partial t}+u\cdot\nabla L_{0}=-\rho_{in}ML_{0}+k_{L_{1}}rL_{1}-\omega_{L_{0}}vL_{0}+D_{l,L_{0}}\nabla^{2}L_{0}$ (A.8)

$\begin{matrix} \frac{dH_{i}}{dt}+u\cdot\nabla H_{i}=k_{H_{i+1}}rH_{i+1}-k_{H_{i}}rH_{i}-\omega_{H_{i}}vH_{i}+\omega_{H_{i-1}}vH_{i-1} \\ +D_{l,H_{i}}\nabla^{2}H_{i} (1\leq i\leq N_{\max,H}) \end{matrix}$ (A.9)

$\frac{dH_{0}}{dt}+u\cdot\nabla H_{0}=k_{H_{1}}rH_{1}-\omega_{H_{0}}vH_{0}+D_{l,H_{0}}\nabla^{2}H_{0}$ (A.10)

$\frac{\partial r}{\partial t}+u\cdot\nabla r=-rd{}_{ox} \left(\sum_{i=1}^{N_{\max,L}} k_{L_{i}}L_{i}+\sum_{i=1}^{N_{\max,H}} k_{H_{i}}H_{i}\right)+D_{l,r}\nabla^{2}r$ (A.11)

$\frac{dv}{dt}+u\cdot\nabla v=-vd_{antox}\left(\sum_{i=0}^{N_{\max,L}-1} \omega_{L_{i}}L_{i}+\sum_{i=0}^{N_{\max,H}-1} \omega_{H_{i}}H_{i}\right)+D_{l,v}\nabla^{2}v$ (A.12)

where $D_{l,L_{i}},D_{l,H_{i}},D_{l,r},D_{l,v}$ are the rates of diffusion of LDL, HDL, radicals and antioxidants in the lumen, respectively. It is to be expected that in the lumen the lower levels of radicals and greater antioxidant content [22] mean that many of the terms involving $k_{l,L_{i}},k_{l,H}$ will be negligible.

Note that in this model the main “source” of LDL and HDL and various other species (e.g., free radical and anti-oxidant) in the intima will be diffusion across the arterial wall, and so dealt with by boundary conditions there. Possibly this will be the case for the “sink” term as well. However, in the lumen it is possible that we shall require source and sink terms for some of these species, in particular the unoxidised HDL and LDL, $H_{N_{\max,H}},L_{N_{\max,L}}$, free radical and anti-oxidant terms, $r,v$, respectively.

**A.2.2 Stage 2 processes (occurring in the intima only)**

The processes occurring exclusively in the intima are those of stages 2 and 3. The stage 2 processes involve cell motion and therefore generally require the concept of chemo­taxis, the movement of cells up a chemical gradient. Chemotaxis has been described mathematically by Keller and Segel [23, 24] and Murray [25]. However the governing equations for chemo-attractant and proliferation molecules do not require this concept and may be modelled by similar types of reaction-diﬀusion processes as those of sec­tion A.2.1. Oxidative modification of lipoproteins, as described in section A.2.1, may result in a variety of products of lipid peroxidation, affecting the arterial wall. Therefore we have:

$\frac{dE_{0}}{dt}=\tau(E_{0ss}-E_{0})-\sum_{i=0}^{N_{\max,L}-1} \iota_{L_{i}}E_{0}L_{i}$ (A.13)

$\frac{dE_{1}}{dt}=\sum_{i=0}^{N_{\max,L}-1} \iota_{L_{i}}E_{0}L_{i}-d_{E}E_{1}$ (A.14)

$\begin{matrix} \frac{\partial C}{\partial t}=\rho_{CE}E_{1}+\rho_{CM}M+\rho_{CT}T+\rho_{CS}S+D_{C}\nabla^{2}C \\ -d_{CM}MC-d_{CT}TC-d_{Cm}mC \end{matrix}$ (A.15)

$\frac{\partial P}{\partial t}=\rho_{PT}T+D_{P}\nabla^{2}P$ (A.16)

$\frac{\partial m}{\partial t}+\nabla\cdot[m\chi_{m}(C)\nabla C]=\mu mP-\rho_{M}mP+D_{m}\nabla^{2}m$ (A.17)

$\frac{\partial M}{\partial t}+\nabla\cdot[M\chi_{M}(C)\nabla C]=\rho_{M}mP-d_{M}(\eta/M)M+D_{M}\nabla^{2}M$ (A.18)

$\frac{\partial\eta}{\partial t}+\nabla\cdot[\eta\chi_{M}(C)\nabla C]=\rho_{in}(\eta/M)ML_{0}-d_{M}(\eta/M)\eta+\nabla\cdot[(\eta/M)D_{M}\nabla M]$ (A.19)

$\frac{\partial T}{\partial t}+\nabla\cdot[T\chi_{T}(C)\nabla C]=-d_{T}T+D_{T}\nabla^{2}T$ (A.20)

where $E_{0},E_{1}$ are the undamaged and damaged EC concentrations, $C$ is the chemo-attractant concentration, $P$ is the proliferation factor, $m$ is the monocyte concentration, $\eta$ is the bound lipid concentration and $T$ is the T-cell concentration. Here $\tau$ is the repopulation rate of ECs, $\iota_{L_{i}}$ are the rates of damage of ECs per unit of LDL concentration with oxidation status $i$, and $E_{0ss}$ is the target EC concentration (a constant). $\rho_{CE},\rho_{CM},\rho_{CT},\rho_{CS}$ are the chemo-attractant production rates due to damaged ECs, macrophages, T-cells and VSMCs, respectively. Our assumption that chemo-attractant is produced by damaged ECs is not made by McKay *et al.* [1]. $\rho_{PT}$ is the proliferation factor production rate per unit of T-lymphocyte concentration, $\rho_{M}$ is the monocyte → macrophage conversion rate, $\mu$ is the monocyte proliferation rate per unit of proliferation factor and $\rho_{in}\equiv\rho_{in}(\eta/M)$ is the bound lipid ingestion rate per unit concentration macrophage and per unit concentration of fully-oxidised LDL. $d_{E}$ is the death/degradation rates of the damaged ECs.

We assume that the bound lipid ingestion rate, $\rho_{in}\equiv\rho_{in}(\eta/M)$, and the macrophage death rate, $d_{M}\equiv d_{M}\left(\eta/M\right)$, are functions solely of the bound lipid/macrophage ratio $\eta/M$. In particular we shall usually assume that the former is some decreasing function of this ratio, and that the latter is some increasing function of this ratio. McKay *et al.* assume that:

$\rho_{in}\left(\eta/M\right)=\frac{\rho_{in,0}}{1+R_{1}\left(\eta/M\right)^{n_{1}}}$ (A.21)

$d_{M}\left(\eta/M\right)=d_{M,0}+R_{2}\left(\eta/M\right)^{n_{2}}$ (A.22)

for some (positive) constants $R_{1},R_{2},n_{1},n_{2}$. $\rho_{in,0}$ is the bound lipid ingestion rate at zero lipid concentration, and $d_{M,0}$ is the rate of macrophage death at zero lipid concentration. The data of Zhao *et al.* [26] suggest a slightly different form for the ingestion function:

$\rho_{in}\left(\eta/M\right)=\rho_{in,high}+[\rho_{in,0}-\rho_{in,high}]\exp[-R_{3}\eta/M]$ (A.23)

Lacking any data on shape of dose response, we shall assume a linear relation between macrophage mortality and bound lipid concentration (i.e. $n_{2}=1$ in (A.22)):

$d_{M}\left(\eta/M\right)=d_{M,0}+R_{2}\eta/M$ (A.24)

**A.2.3 Stage 3 processes (occurring in the intima only)**

These processes occur exclusively in the intima and again require the concept of chemotaxis, at least for modelling of VSMC ($S$). Therefore we have the following sets of reaction diffusion equations:

$\frac{\partial S}{\partial t}+\nabla\cdot[S\chi_{S}(C)\nabla C]=-d_{S}S-J_{mech}S+D_{S}\nabla^{2}S$ (A.25)

$\frac{\partial G}{\partial t}=-\left[d_{GT}T+d_{GM}M\right]G+\rho_{G}S$ (A.26)

$\frac{\partial N}{\partial t}=d_{M}(\eta/M)[\eta+d_{MM}M]+d_{S}d_{SS}S+d_{T}d_{TT}T$ (A.27)

where $S$ is the VSMC concentration, $G$ is the collagen concentration and $N$ is the necrotic core concentration. Here $\rho_{G}$ is the collagen augmentation rate per unit of VSMC concentration. $J_{mech}$ is the degradation rate of VSMC due to mechanical stress. $d_{S},d_{T}$ are the rates of augmentation of the necrotic core (by dying VSMCs and T-cells respectively), and $d_{GT},d_{GM}$ are the destruction/degradation rates of collagen per unit of T cells and per unit of macrophages. It should be noted that the necrotic core is also augmented by dying macrophages (the $d_{M}(\eta/M)d_{MM}M$ term in equation (A.27)) and by released (non-bound) lipid (the $d_{M}(\eta/M)\eta$ term in equation (A.27): see equation (A.19)). $d_{SS},d_{TT}$ are the multipliers of VSMC and T-cell death for volume of necrotic core. Also, $\chi_{S}(C)$ is the chemotactic factor associated with VSMC.

**References**

1. McKay C, McKee S, Mottram N, Mulholland T, Wilson S *et al.* Towards a model of atherosclerosis (2005) Strathclyde Mathematics Research Report (March 2005), No. 4.

2. Ross R (1999) Atherosclerosis is an inflammatory disease. *Am. Heart J.* **138**: S419-420.

3. Caro C, Fitzgerald G, Schroter R (1971) Atheroma and arterial wall shear: Observation, correlation and proposal of a shear dependent mass transfer mechanism for atherogenesis. *Proc. Royal Soc. London Biol.* *Sci.* **177**: 109–159.

4. Lever MJ, Jay MT (1993) Connective and diffusive transport of plasma proteins across the walls of large blood vessels. *Front. Med. Biol. Eng.* **5**: 45-50.

5. Esterbauer H, Gebicki J, Puhl H, Jurgens G (1992) Review Article: The role of lipid peroxidation and antioxidants in oxidative modification of LDL. *Free Radic. Biol. Med.* **13**: 341–390.

6. Stocker R (1999) The ambivalence of vitamin E in atherogenesis. *Trends Biochem. Sci.* **24**: 219–223.

7. Heart Protection Study Collaborative Group (2002) MRC/BHF Heart Protection Study of cholesterol lowering with simuvastatin in 20,536 high risk individuals: a randomized placebo-controlled study. *Lancet* **360**: 7-22.

8. Farmer JA, Torre-Amione G (2002) Atherosclerosis and inflammation, *Curr. Atheroscler. Rep.* **4**: 92-98.

9. van Lenten BJ, Navab M, Shih D (2001) The role of high density lipoproteins in oxidation and inflammation. *Trends Cardiovasc. Med.* **11**: 151-161.

10. Jones GE (2000) Cellular signalling in macrophage migration and chemotaxis. *J. Leuk. Biology* **68**: 593-602.

11. Yang LV, Radu CG, Wang L, Riedinger M, Witte ON (2005) Gi-independent macrophage chemotaxis to lysophosphatidylcholine via the immunoregulatory GPCR G2A. *Blood* **105**: 1127-1134.

12. Hajjar DP, Haberland ME (1997) Lipoprotein trafficking in vascular cells. *J. Biol. Chem.* **272**: 22975-22978.

13. Goldstein JL, Ho YK, Basu SK, Brown MS (1979) Binding site on macrophages that mediates uptake and degradation of acetylated low density lipoproteins, producing massive cholesterol deposition. *Proc. Natl Acad. Sci. USA* **76**: 333-337.

14. Braganza DM, Bennett MR (2001) New insights into atherosclerotic plaque rupture. *Postgrad. Med. J.* **77**: 94-98.

15. Clarke MC, Littlewood TD, Figg N, Maguire JJ, Davenport AP *et al.* (2008) Chronic apoptosis of vascular smooth muscle cells accelerates atherosclerosis and promotes calcification and medial degeneration. *Circ. Res.* **102**: 1529-1538.

16. Reape T, Groot P (1999) Chemokines and atherosclerosis. *Atherosclerosis* **147**: 213–225.

17. Ross R (1995) Cell biology of atherosclerosis. *Annual Rev. Physiol.* **57**: 791–804.

18. Li C, Xu Q (2000) Mechanical stress-initiated signal transductions in vas­cular smooth muscle cells. *Cellular Signalling* **12**: 435–445.

19. Wernig F, Xu Q (2002) Mechanical stress-induced apoptosis in the cardio­vascular system. *Prog. Biophys. Molec. Biol.* **78**: 105–137.

20. Libby P (2002) Atherosclerosis: The new view. *Scientiﬁc American* **286**: 29–37.

21. Tricot O, Mallat Z, Heymes C, Belmin J, Leseche G *et al.* (2000) Relation between endothelial cell apoptosis and blood ﬂow direction in human atherosclerosis. *Circulation* **101**: 2450–2453.

22. Cobbold C, Sherratt J, Maxwell S (2002) Lipoprotein oxidation and its signiﬁcance for atherosclerosis: a mathematical approach. *Bull. Math­. Biol.* **64**: 65–95.

23. Keller EF, Segel LA (1971) Model for chemotaxis. *J. Theoret. Biol.* **30**: 225–234.

24. Keller EF, Segel LA (1971) Traveling bands of chemotactic bacteria: a theoretical analysis. *J. Theoret. Biol.* **30**: 235–248.

25. Murray J (1990) *Mathematical Biology: Volume I.* Springer-Verlag, New York.

26. Zhao B, Li Y, Buono C, Waldo SW, Jones NL *et al.* (2006) Constitutive receptor-independent low density lipoprotein uptake and cholesterol accumulation by macrophages differentiated from human monocytes with macrophage-colony-stimulating factor (M-CSF). *J. Biol. Chem.* **281**: 15757-15762.

27. Zhang Y, Bissing JW, Xu L, Ryan AJ, Martin SM *et al.* (2001) Nitric oxide synthase inhibitors decrease coronary sinus-free radical concentration and ameliorate myocardial stunning in an ischemia-reperfusion model. *J. Am. Coll. Cardiol.* **38**: 546-54.

28. Boisvert WA, Santiago R, Curtiss LK, Terkeltaub RA (1998) A leukocyte homologue of the IL-8 receptor CXCR-2 mediates the accumulation of macrophages in atherosclerotic lesions of LDL receptor-deficient mice. *J. Clin. Invest.* **101**: 353-363.

29. Libby P (2002) Inflammation in atherosclerosis. *Nature* **420**: 868-874.

30. Haraoka S, Shimokama T, Watanabe T (1997) Role of T lymphocytes in the pathogenesis of atherosclerosis: animal studies using athymic nude rats. *Ann. N.Y. Acad. Sci.* **811**: 515-518.

31. Pierce JH, Di Marco E, Cox GW, Lombardi D, Ruggiero M *et al.* (1990) Macrophage-colony-stimulating factor (CSF-1) induces proliferation, chemotaxis, and reversible monocytic differentiation in myeloid progenitor cells transfected with the human c-*fms*/CSF-1 receptor cDNA. *Proc. Natl Acad. Sci. USA* **87**: 5613-5617.

32. Ingold KU, Bowry VW, Stocker R, Walling C (1993) Autoxidation of lipids and antioxidation by α-tocopherol and ubiquinol in homogeneous solution and in aqueous dispersions of lipids: unrecognised consequences of lipid particle size as exemplified by oxidation of human low density lipoprotein. *Proc. Natl. Acad. Sci. USA* **90**: 45–49.

33. Packer JE, Slater TF, Willson RL (1979) Direct observation of a free radical interaction between vitamin E and vitamin C. *Nature* **278**: 737–738.

34. Jialal I, Vega GL, Grundy SM (1990) Physiologic levels of ascorbate inhibit the oxidative modification of low density lipoprotein. *Atherosclerosis* **82**: 185–191.

35. Crouse JR, Parks JS, Schey HM, Kahl FR (1985) Studies of low density lipoprotein molecular weight in human beings with coronary artery disease. *J. Lipid Res.* **26**: 566-574.

36. Schwegler JS, Knorz MC, Akkoyun I, Liesenhoff H (1997) Basic, not acidic fibroblast growth factor stimulates proliferation of cultured human retinal pigment epithelial cells. [*Mol. Vis.*](javascript:AL_get(this, 'jour', 'Mol Vis.');) **3**: 10.

37. Sachs RK, Brenner DJ (2005) Solid tumor risks after high doses of ionizing radiation. *Proc. Natl Acad. Sci. USA* **102**: 13040-13045.

38. Thomas JP, Geiger PG, Girotti AW (1993) Lethal damage to endothelial cells by oxidized low density lipoprotein: role of selenoperoxidases in cytoprotection against lipid hydroperoxide- and iron-mediated reactions. *J. Lipid Res.* **34**: 479-490.

39. Liu B, Sidiropoulos A, Zhao B, Dierichs R (1998) Oxidized LDL damages endothelial cell monolayer and promotes thrombocyte adhesion. *Am. J. Hematol.* **57**: 341-343.

40. Steffen Y, Schewe T, Sies H (2005) Epicatechin protects endothelial cells against oxidized LDL and maintains NO synthase. [*Biochem. Biophys. Res. Commun.*](javascript:AL_get(this, 'jour', 'Biochem Biophys Res Commun.');) **331**: 1277-1283.

41. McGeachie J (2008) Blue histology: more about endothelial cells. <http://www.lab.anhb.uwa.edu.au/mb140/MoreAbout/Endothel.htm>.

42. Filler SG, Pfunder AS, Spellberg BJ, Spellberg JP, Edwards JE Jr (1996) Candida albicans stimulates cytokine production and leukocyte adhesion molecule expression by endothelial cells. *Infect. Immun.* **64**: 2609-2617.

43. Bosco MC, Puppo M, Pastorino S, Mi Z, Melillo G *et al.* (2004) Hypoxia selectively inhibits monocyte chemoattractant protein-1 production by macrophages. *J. Immunol.* **172**: 1681-1690.

44. Yoo JK, Kwon H, Khil LY, Zhang L, Jun HS *et al.* (2005) IL-18 induces monocyte chemotactic protein-1 production in macrophages through the phosphatidylinositol 3-kinase/Akt and MEK/ERK1/2 pathways. *J. Immunol.* **175**: 8280-8286.

45. Villiger PM, Cronin MT, Amenomori T, Wachsman W, Lotz M (1991) IL-6 production by human T lymphocytes. Expression in HTLV-1-infected but not in normal T cells. *J. Immunol.* **146**: 550-559.

46. Lukacs NW, Chensue SW, Karpus WJ, Lincoln P, Keefer C *et al.* (1997) C-C chemokines differentially alter interleukin-4 production from lymphocytes. *Am. J. Pathol.* **150**: 1861-1868.

47. Tang S, Liu H, Chen G, Rao Q, Geng Y *et al.* (2000) Internalization and half-life of membrane-bound macrophage colony-stimulating factor. *Chinese Sci. Bull.* **45**: 1697-1703.

48. Knowles DM (ed.) (2000) *Neoplastic hematopathology* (2^nd^ ed.) Lippincott Williams & Wilkins.

49. Frétier S, Besse A, Delwail A, Garcia M, Morel F *et al.* (2002) Cyclosporin A inhibition of macrophage colony-stimulating factor (M-CSF) production by activated human T lymphocytes. *J. Leuk. Biol.* **71**: 289-294.

50. Becker S, Warren MK, Haskill S (1987) Colony-stimulating factor-induced monocyte survival and differentiation into macrophages in serum-free cultures. *J. Immunol.* **139**: 3703-3709.

51. Dimmeler S, Haendeler J, Galle J, Zeiher AM (1997) Oxidized low-density lipoprotein induces apoptosis of human endothelial cells by activation of CPP32-like proteases. A mechanistic clue to the 'response to injury' hypothesis. *Circulation* **95**: 1760-1763.

52. Asquith B, Zhang Y, Mosley AJ, de Lara CM, Wallace DL *et al.* (2007) *In vivo* T lymphocyte dynamics in humans and the impact of human T-lymphotropic virus 1 infection. *Proc. Natl Acad. Sci. USA* **104**: 8035-8040.

53. Hanson CA (2001) Peripheral blood and bone marrow: morphology, counts and differentials, and reactive disorders. pp.797-829 in: McClatchey KD (ed). *Clinical laboratory medicine*, Lippincott, Williams and Wilkins.

54. Kellner-Weibel G, Jerome WG, Small DM, Warner GJ, Stoltenborg JK *et al.* (1998) Effects of intracellular free cholesterol accumulation on macrophage viability: a model for foam cell death. *Arterioscler. Thromb. Vasc. Biol.* **18**: 423-431.

55. Takaku M, Wada Y, Jinnouchi K, Takeya M, Takahashi K *et al.* (1999) An in vitro coculture model of transmigrant monocytes and foam cell formation. *Arterioscler. Thromb. Vasc. Biol.* **19**: 2330-2339.

56. Klouche M, May AE, Hemmes M, Messner M, Kanse SM *et al.* (1999) Enzymatically modified, nonoxidized LDL induces selective adhesion and transmigration of monocytes and T-lymphocytes through human endothelial cell monolayers. *Arterioscler. Thromb. Vasc. Biol.* **19**: 784-793.

57. Han KH, Tangirala RK, Green SR, Quehenberger O (1998) Chemokine receptor CCR2 expression and monocyte chemoattractant protein-1-mediated chemotaxis in human monocytes. A regulatory role for plasma LDL. *Arterioscler. Thromb. Vasc. Biol.* **18**: 1983-1991.

58. Seet BT, McCaughan CA, Handel TM, Mercer A, Brunetti C *et al.* (2003) Analysis of an orf virus chemokine-binding protein: Shifting ligand specificities among a family of poxvirus viroceptors. *Proc. Natl Acad. Sci. USA* **100**: 15137-15142; **101**: 5180.

59. Cullen JP, Morrow D, Jin Y, von Offenberg Sweeney N, Sitzmann JV *et al.* (2007) Resveratrol inhibits expression and binding activity of the monocyte chemotactic protein-1 receptor, CCR2, on THP-1 monocytes. *Atherosclerosis* **195**: e125-e133.

60. Krombach F, Münzing S, Allmeling AM, Gerlach JT, Behr J *et al.* (1997) Cell size of alveolar macrophages: an interspecies comparison. *Environ. Health Perspect.* **105** Suppl 5: 1261-1263.

61. Cheung RK, Grinstein S, Dosch HM, Gelfand EW (1982) Volume regulation by human lymphocytes: characterization of the ionic basis for regulatory volume decrease. *J. Cell. Physiol.* **112**: 189-196.

62. Atmeh RF (1990) Isolation and identification of HDL particles of low molecular weight. *J. Lipid Res.* **31**: 1771-1780.

63. Chandrasekhar S (1943) Stochastic problems in physics and astronomy. *Rev. Mod. Phys.* **15**: 1-89.

64. Suzu S, Ohtsuki T, Yanai N, Takatsu Z, Kawashima T *et al.* (1992) Identification of a high molecular weight macrophage colony-stimulating factor as a glycosaminoglycan-containing species. *J. Biol. Chem.* **267**: 4345-4348.

65. Lund O, Hansen J, Mosekilde E, Nielsen J-O, Stig-Hansen J-E (1993) A model of enhancement and inhibition of HIV infection of monocytes by antibodies against HIV. *J. Biol. Phys.* **19**: 133-145.

66. Shin S, Ku Y, Park M-S, Suh J-S (2004) Measurement of red cell deformability and whole blood viscosity using laser-diffraction slit rheometer. *Korea-Australia Rheology J.* **16**, 85-90.

67. Lauffenburger D, Rothman C, Zigmond SH (1983) Measurement of leukocyte motility and chemotaxis parameters with a linear under-agarose migration assay. *J. Immunol.* **131**: 940-947.

68. Napoli C, D'Armiento FP, Mancini FP, Postiglione A, Witztum JL *et al.* (1997) Fatty streak formation occurs in human fetal aortas and is greatly enhanced by maternal hypercholesterolemia. Intimal accumulation of low density lipoprotein and its oxidation precede monocyte recruitment into early atherosclerotic lesions. *J. Clin. Invest.* **100**: 2680-2690.

69. Bing H, Wang J, Zhang C, Cai H (2004) Positive correlation between in vivo oxidized LDL and LDL immune complexes. *Clin. Biochem.* **37**: 72-75.

70. Henry JA, Bolla M, Osmond C, Fall C, Barker DJ *et al.* (1997) The effects of genotype and infant weight on adult plasma levels of fibrinogen, factor VII, and LDL cholesterol are additive. *J. Med. Genet.* **34**: 553-558.

71. Cannon JG, Sabatier MJ, Marinik EL, Schwark EH, Haddow S *et al.* (2009) Femoral artery diameter and arteriogenic cytokines in healthy women. *Vascul. Pharmacol.* **50**: 104-109.

72. Navab M, Hough GP, Stevenson LW, Drinkwater DC, Laks H *et al.* (1988) Monocyte migration into the subendothelial space of a coculture of adult human aortic endothelial and smooth muscle cells. *J. Clin. Invest.* **82**: 1853-1863.

73. Lichtman MA, Beutler E, Kaushansky K, Kipps TJ, Seligsohn U *et al.* (2005) *Williams Hematology (7th Ed)* McGraw-Hill Medical.

74. Gordon MY (1975) A comparison of the radiosensitivity and O.E.R. of human and mouse marrow progenitor cells cultured in agar in diffusion chambers. *Int. J. Radiat. Biol.* **28**: 285-290.

75. Deschavanne PJ, Fertil B (1996) A review of human cell radiosensitivity *in vitro*. *International Journal of Radiat. Oncol. Biol. Phys.* **34**: 251-266.

76. Leung WH, Stadius ML, Alderman EL (1991) Determinants of normal coronary artery dimensions in humans. *Circulation* **84**: 2294-2306.

77. Terry JG, Carr JJ, Tang R, Evans GW, Kouba EO *et al.* (2005) Coronary artery calcium outperforms carotid artery intima-media thickness as a noninvasive index of prevalent coronary artery stenosis. *Arterioscler. Thromb. Vasc. Biol.* **25**: 1723-1728.

**Supplementary material B.**

**Spatially averaged solutions of perturbed equilibrium solution from simplified reaction-diffusion model in main text**

We present solutions to the perturbation equations (42)-(48) in various cases. In general we have:

$\begin{matrix} \int_{\Omega_{I}} C_{\Delta}(t)dx=\int_{\Omega_{I}} C_{\Delta}(0)dx+\rho_{CE}\int_{0}^{t} ds\int_{\Omega_{I}} E_{1,\Delta}(s)dx \text{if }m_{eq}=0 \\ +[\rho_{CM}-d_{CM}C_{eq}]\left\{\begin{matrix} \frac{[1-\exp[-d_{M}(0)t]]\int_{\Omega_{I}} M_{\Delta}(0)dx}{d_{M}(0)} \\ +\frac{\rho_{M}P_{eq}\int_{\Omega_{I}} m_{\Delta}(0)dx}{d_{M}(0)+P_{eq}[\mu-\rho_{M}]}\left\{\begin{matrix} \frac{\exp[P_{eq}[\mu-\rho_{M}]t]-1}{P_{eq}[\mu-\rho_{M}]} \\ +\frac{\exp[-d_{M}(0)t]\text{-1}}{d_{M}(0)} \end{matrix}\right\} \end{matrix}\right\} \\ +\frac{[\rho_{CT}-d_{CT}C_{eq}][1-\exp[-d_{T}t]]\int_{\Omega_{I}} T_{\Delta}(0)dx}{d_{T}} \\ -\frac{d_{Cm}C_{eq}[\exp[P_{eq}[\mu-\rho_{M}]t]-1]\int_{\Omega_{I}} m_{\Delta}(0)dx}{P_{eq}[\mu-\rho_{M}]} \\ \end{matrix}$ (B.1a)

$$\begin{matrix} \int_{\Omega_{I}} C_{\Delta}(t)dx=\exp[-d_{Cm}m_{eq}t]\int_{\Omega_{I}} C_{\Delta}(0)dx+\rho_{CE}\int_{0}^{t} \exp[d_{Cm}m_{eq}(s-t)]ds\int_{\Omega_{I}} E_{1,\Delta}(s)dx\text{ if }P_{eq}=0 \\ +[\rho_{CM}-d_{CM}C_{eq}]\left\{\begin{matrix} \frac{[\exp[-d_{M}(0)t]-\exp[-d_{Cm}m_{eq}t]]\int_{\Omega_{I}} M_{\Delta}(0)dx}{d_{Cm}m_{eq}-d_{M}(0)}+ \\ \rho_{M}m_{eq}\left\{\begin{matrix} \frac{\left(\int_{\Omega_{I}} P_{\Delta}(0)dx+\frac{\rho_{PT}}{d_{T}}\int_{\Omega_{I}} T_{\Delta}(0)dx\right)}{d_{M}(0)d_{Cm}m_{eq}}[\text{1-}\exp[-d_{Cm}m_{eq}t]] \\ -\frac{\left(\int_{\Omega_{I}} P_{\Delta}(0)dx+\frac{\rho_{PT}}{d_{T}}\int_{\Omega_{I}} T_{\Delta}(0)dx\right)}{d_{M}(0)[d_{Cm}m_{eq}-d_{M}(0)]}\left[\begin{matrix} \exp[-d_{M}(0)t] \\ \text{-}\exp[-d_{Cm}m_{eq}t] \end{matrix}\right] \\ -\frac{\rho_{PT}\int_{\Omega_{I}} T_{\Delta}(0)dx}{d_{T}[d_{M}(0)-d_{T}]}\left[\begin{matrix} \frac{\exp[-d_{T}t]-\exp[-d_{Cm}m_{eq}t]}{d_{Cm}m_{eq}-d_{T}} \\ -\frac{\exp[-d_{M}(0)t]-\exp[-d_{Cm}m_{eq}t]}{d_{Cm}m_{eq}-d_{M}(0)} \end{matrix}\right] \end{matrix}\right\} \end{matrix}\right\} \\ +\frac{[\rho_{CT}-d_{CT}C_{eq}][\exp[-d_{T}t]-\exp[-d_{Cm}m_{eq}t]]\int_{\Omega_{I}} T_{\Delta}(0)dx}{[d_{Cm}m_{eq}-d_{T}]} \\ -d_{Cm}C_{eq}\left\{\begin{matrix} \left(\int_{\Omega_{I}} m_{\Delta}(0)dx-\frac{m_{eq}[\mu-\rho_{M}]\rho_{PT}\int_{\Omega_{I}} T_{\Delta}(0)dx}{d_{T}^{2}}\right)\frac{[1-\exp[-d_{Cm}m_{eq}t]]}{d_{Cm}m_{eq}} \\ +m_{eq}[\mu-\rho_{M}]\left(\int_{\Omega_{I}} P_{\Delta}(0)dx+\frac{\rho_{PT}}{d_{T}}\int_{\Omega_{I}} T_{\Delta}(0)dx\right)\left(\begin{matrix} \frac{t}{d_{Cm}m_{eq}} \\ -\frac{[1-\exp[-d_{Cm}m_{eq}t]]}{d_{Cm}^{2}m_{eq}^{2}} \end{matrix}\right) \\ +\frac{\rho_{PT}m_{eq}[\mu-\rho_{M}][\exp[-d_{T}t]-\exp[-d_{Cm}m_{eq}t]]\int_{\Omega_{I}} T_{\Delta}(0)dx}{d_{T}^{2}[d_{Cm}m_{eq}-d_{T}]} \end{matrix}\right\} \\ \end{matrix}$$

(B.1b)

$\int_{\Omega_{I}} P_{\Delta}(t)dx=\int_{\Omega_{I}} P_{\Delta}(0)dx+\frac{\rho_{PT}}{d_{T}}[1-\exp[-d_{T}t]]\int_{\Omega_{I}} T_{\Delta}(0)dx$ (B.2)

$\int_{\Omega_{I}} m_{\Delta}(t)dx=\left\{\begin{matrix} \exp[P_{eq}[\mu-\rho_{M}]t]\int_{\Omega_{I}} m_{\Delta}(0)dx\text{ if }m_{eq}=0 \\ \int_{\Omega_{I}} m_{\Delta}(0)dx+m_{eq}[\mu-\rho_{M}]\left\{\begin{matrix} t\left(\int_{\Omega_{I}} P_{\Delta}(0)dx+\frac{\rho_{PT}}{d_{T}}\int_{\Omega_{I}} T_{\Delta}(0)dx\right) \\ -\frac{\rho_{PT}}{d_{T}^{2}}[1-\exp[-d_{T}t]]\int_{\Omega_{I}} T_{\Delta}(0)dx \end{matrix}\right\} \end{matrix}\right.\text{ if }P_{eq}=0$ (B.3)

$\int_{\Omega_{I}} M_{\Delta}(t)dx=\left\{\begin{matrix} \begin{matrix} \exp[-d_{M}(0)t]\int_{\Omega_{I}} M_{\Delta}(0)dx \\ +\frac{\rho_{M}P_{eq}}{d_{M}(0)+P_{eq}[\mu-\rho_{M}]}\left[\begin{matrix} \exp[P_{eq}[\mu-\rho_{M}]t] \\ \text{-}\exp[-d_{M}(0)t] \end{matrix}\right]\int_{\Omega_{I}} m_{\Delta}(0)dx\text{ if }m_{eq}=0 \end{matrix} \\ \begin{matrix} \exp[-d_{M}(0)t]\int_{\Omega_{I}} M_{\Delta}(0)dx \\ +\rho_{M}m_{eq}\left\{\begin{matrix} \frac{\left[\text{1-}\exp[-d_{M}(0)t]\right]}{d_{M}(0)}\left(\int_{\Omega_{I}} P_{\Delta}(0)dx+\frac{\rho_{PT}}{d_{T}}\int_{\Omega_{I}} T_{\Delta}(0)dx\right) \\ -\frac{\rho_{PT}\left[\exp[-d_{T}t]-\exp[-d_{M}(0)t]\right]}{d_{T}[d_{M}(0)-d_{T}]}\int_{\Omega_{I}} T_{\Delta}(0)dx \end{matrix}\right\} \end{matrix} \end{matrix}\right.\text{ if }P_{eq}=0$ (B.4)

$\begin{matrix} \int_{\Omega_{I}} \eta_{\Delta}(t)dx=\exp[-d_{M}(0)t]\int_{\Omega_{I}} \eta_{\Delta}(0)dx \\ +\rho_{in}(0)\int_{0}^{t} \exp[d_{M}(0)[s-t]]ds\int_{\Omega_{I}} M_{\Delta}(s)L_{0}(s)dx \end{matrix}$ (B.5)

$\int_{\Omega_{I}} T_{\Delta}(t)dx=\exp[-d_{T}t]\int_{\Omega_{I}} T_{\Delta}(0)dx$ (B.6)

$\begin{matrix} \int_{\Omega_{I}} N_{\Delta}(t)dx=\int_{\Omega_{I}} N_{\Delta}(0)dx+d_{M}(0)[\int_{0}^{t} ds\int_{\Omega_{I}} \eta_{\Delta}(s)dx+d_{MM}\int_{0}^{t} ds\int_{\Omega_{I}} M_{\Delta}(s)dx] \\ +d_{TT}[1-\exp[-d_{T}t]]\int_{\Omega_{I}} T_{\Delta}(0)dx \end{matrix}$ (B.7)

It is immediate from equations (31)-(32) that:

$E_{0,\Delta}(t)=E_{0,\Delta}(0)\exp\left[-\int_{0}^{t} \left[\tau+\sum_{i=0}^{N_{\max,L}-1} \iota_{L_{i}}L_{i}(s)\right]ds\right]$ (B.8)

$\begin{matrix} E_{1,\Delta}(t)=E_{1,\Delta}(0)\exp[-d_{E}t]+E_{0,\Delta}(0)\int_{0}^{t} \left(\sum_{i=0}^{N_{\max,L}-1} \iota_{L_{i}}L_{i}(s)\right)\exp[d_{E}(s-t)]\exp\left[-\int_{0}^{s} \left[\tau+\sum_{i=0}^{N_{\max,L}-1} \iota_{L_{i}}L_{i}(w)\right]dw\right]ds \\ =E_{1,\Delta}(0)\exp[-d_{E}t]+E_{0,\Delta}(0)\left\{\begin{matrix} \exp[-d_{E}t]-\exp\left[-\int_{0}^{t} \left[\tau+\sum_{i=0}^{N_{\max,L}-1} \iota_{L_{i}}L_{i}(w)\right]dw\right] \\ +[d_{E}-\tau]\int_{0}^{t} \exp[d_{T}(s-t)]\exp\left[-\int_{0}^{s} \left[\tau+\sum_{i=0}^{N_{\max,L}-1} \iota_{L_{i}}L_{i}(w)\right]dw\right]ds \end{matrix}\right\} \end{matrix}$ (B.9)

and when, further, all $L_{i}(x,t)\equiv L_{i}$ ($0\leq i\leq N_{\max,L}-1$) are constant over space and time (B.8) and (B.9) reduce to:

$E_{0,\Delta}(t)=E_{0,\Delta}(0)\exp\left[-\left[\tau+\sum_{i=0}^{N_{\max,L}-1} \iota_{L_{i}}L_{i}\right]t\right]$ (B.8’)

$E_{1,\Delta}(t)=E_{1,\Delta}(0)\exp[-d_{E}t]+E_{0,\Delta}(0)\frac{\sum_{i=0}^{N_{\max,L}-1} \iota_{L_{i}}L_{i}}{\tau-d_{E}+\sum_{i=0}^{N_{\max,L}-1} \iota_{L_{i}}L_{i}}\left[\begin{matrix} \exp[-d_{E}t] \\ -\exp\left[-\left(\tau+\sum_{i=0}^{N_{\max,L}-1} \iota_{L_{i}}L_{i}\right)t\right] \end{matrix}\right]$ (B.9’)

and we can then write analytic forms of (B.5), (B.7):

$\begin{matrix} \int_{\Omega_{I}} \eta_{\Delta}(t)dx= \\ \left\{\begin{matrix} \begin{matrix} \exp[-d_{M}(0)t]\int_{\Omega_{I}} \eta_{\Delta}(0)dx \text{if }m_{eq}=0 \\ +\rho_{in}(0)L_{0}\left\{\begin{matrix} t\exp[-d_{M}(0)t]\int_{\Omega_{I}} M_{\Delta}(0)dx \\ +\frac{\rho_{M}P_{eq}\int_{\Omega_{I}} m_{\Delta}(0)dx}{d_{M}(0)+P_{eq}[\mu-\rho_{M}]}\left(\begin{matrix} \frac{\left[\begin{matrix} \exp[P_{eq}[\mu-\rho_{M}]t] \\ \text{-}\exp[-d_{M}(0)t] \end{matrix}\right]}{d_{M}(0)+P_{eq}[\mu-\rho_{M}]} \\ -t\exp[-d_{M}(0)t] \end{matrix}\right) \end{matrix}\right\} \end{matrix} \\ \begin{matrix} \exp[-d_{M}(0)t]\int_{\Omega_{I}} \eta_{\Delta}(0)dx \text{if }P_{eq}=0 \\ +\rho_{in}(0)L_{0}\left\{\begin{matrix} t\exp[-d_{M}(0)t]\int_{\Omega_{I}} M_{\Delta}(0)dx \\ +\frac{\rho_{M}m_{eq}\left(\int_{\Omega_{I}} P_{\Delta}(0)dx+\frac{\rho_{PT}}{d_{T}}\int_{\Omega_{I}} T_{\Delta}(0)dx\right)}{d_{M}(0)}\left(\begin{matrix} \frac{\left[1\text{-}\exp[-d_{M}(0)t]\right]}{d_{M}(0)} \\ -t\exp[-d_{M}(0)t] \end{matrix}\right) \\ -\frac{\rho_{M}m_{eq}\rho_{PT}\int_{\Omega_{I}} T_{\Delta}(0)dx}{d_{T}[d_{M}(0)-d_{T}]}\left(\begin{matrix} \frac{\left[\exp[-d_{T}t]\text{-}\exp[-d_{M}(0)t]\right]}{d_{M}(0)-d_{T}} \\ -t\exp[-d_{M}(0)t] \end{matrix}\right) \end{matrix}\right\} \end{matrix} \end{matrix}\right. \end{matrix}$ (B.5’)

(B.7’)

If we assume that $P_{eq}=0$ and that $L_{0}(x,t)\equiv L_{0}$ is constant over space and time then:

$\begin{matrix} \int_{\Omega_{I}} C_{\Delta}(t)dx=\exp[-d_{Cm}m_{eq}t]\int_{\Omega_{I}} C_{\Delta}(0)dx \\ +\rho_{CE}\left\{\begin{matrix} \frac{[\exp[-d_{E}t]-\exp[-d_{Cm}m_{eq}t]]\int_{\Omega_{I}} E_{1,\Delta}(0)dx}{d_{Cm}m_{eq}-d_{E}} \\ +\frac{\left[\exp\left[-t\left(\tau+\sum_{i=0}^{N_{\max,L}-1} \iota_{L_{i}}L_{i}\right)\right]-\exp[-d_{Cm}m_{eq}t]\right]\left(\sum_{i=0}^{N_{\max,L}-1} \iota_{L_{i}}L_{i}\right)\int_{\Omega_{I}} E_{0,\Delta}(0)dx}{\left(d_{E}-\tau-\sum_{i=0}^{N_{\max,L}-1} \iota_{L_{i}}L_{i}\right)\left(d_{Cm}m_{eq}-\tau-\sum_{i=0}^{N_{\max,L}-1} \iota_{L_{i}}L_{i}\right)} \\ -\frac{[\exp[-d_{E}t]-\exp[-d_{Cm}m_{eq}t]]\left(\sum_{i=0}^{N_{\max,L}-1} \iota_{L_{i}}L_{i}\right)\int_{\Omega_{I}} E_{0,\Delta}(0)dx}{\left(d_{E}-\tau-\sum_{i=0}^{N_{\max,L}-1} \iota_{L_{i}}L_{i}\right)\left(d_{Cm}m_{eq}-d_{E}\right)} \end{matrix}\right\} \end{matrix}$ $\begin{matrix} +[\rho_{CM}-d_{CM}C_{eq}]\left\{\begin{matrix} \frac{[\exp[-d_{M}(0)t]-\exp[-d_{Cm}m_{eq}t]]\int_{\Omega_{I}} M_{\Delta}(0)dx}{d_{Cm}m_{eq}-d_{M}(0)}+ \\ \rho_{M}m_{eq}\left\{\begin{matrix} \frac{\left(\int_{\Omega_{I}} P_{\Delta}(0)dx+\frac{\rho_{PT}}{d_{T}}\int_{\Omega_{I}} T_{\Delta}(0)dx\right)}{d_{M}(0)d_{Cm}m_{eq}}[\text{1-}\exp[-d_{Cm}m_{eq}t]] \\ -\frac{\left(\int_{\Omega_{I}} P_{\Delta}(0)dx+\frac{\rho_{PT}}{d_{T}}\int_{\Omega_{I}} T_{\Delta}(0)dx\right)}{d_{M}(0)[d_{Cm}m_{eq}-d_{M}(0)]}\left[\begin{matrix} \exp[-d_{M}(0)t] \\ \text{-}\exp[-d_{Cm}m_{eq}t] \end{matrix}\right] \\ -\frac{\rho_{PT}\int_{\Omega_{I}} T_{\Delta}(0)dx}{d_{T}[d_{M}(0)-d_{T}]}\left[\begin{matrix} \frac{\exp[-d_{T}t]-\exp[-d_{Cm}m_{eq}t]}{d_{Cm}m_{eq}-d_{T}} \\ -\frac{\exp[-d_{M}(0)t]-\exp[-d_{Cm}m_{eq}t]}{d_{Cm}m_{eq}-d_{M}(0)} \end{matrix}\right] \end{matrix}\right\} \end{matrix}\right\} \\ +\frac{[\rho_{CT}-d_{CT}C_{eq}][\exp[-d_{T}t]-\exp[-d_{Cm}m_{eq}t]]\int_{\Omega_{I}} T_{\Delta}(0)dx}{[d_{Cm}m_{eq}-d_{T}]} \\ -d_{Cm}C_{eq}\left\{\begin{matrix} \left(\int_{\Omega_{I}} m_{\Delta}(0)dx-\frac{m_{eq}[\mu-\rho_{M}]\rho_{PT}\int_{\Omega_{I}} T_{\Delta}(0)dx}{d_{T}^{2}}\right)\frac{[1-\exp[-d_{Cm}m_{eq}t]]}{d_{Cm}m_{eq}} \\ +m_{eq}[\mu-\rho_{M}]\left(\int_{\Omega_{I}} P_{\Delta}(0)dx+\frac{\rho_{PT}}{d_{T}}\int_{\Omega_{I}} T_{\Delta}(0)dx\right)\left(\begin{matrix} \frac{t}{d_{Cm}m_{eq}} \\ -\frac{[1-\exp[-d_{Cm}m_{eq}t]]}{d_{Cm}^{2}m_{eq}^{2}} \end{matrix}\right) \\ +\frac{\rho_{PT}m_{eq}[\mu-\rho_{M}][\exp[-d_{T}t]-\exp[-d_{Cm}m_{eq}t]]\int_{\Omega_{I}} T_{\Delta}(0)dx}{d_{T}^{2}[d_{Cm}m_{eq}-d_{T}]} \end{matrix}\right\} \\ \end{matrix}$ (B.1b’)

Clearly, from (B.8) and (B.9) if $\tau>0$ and $d_{E}>0$ then:

$\lim_{t\to\infty}E_{0,\Delta}(t)=0$ and $\lim_{t\to\infty}E_{1,\Delta}(t)=0$ (B.10)

If we assume that $P_{eq}=0$ and that $L_{0}(x,t)\equiv L_{0}$ is constant over space and time then from (B.1b’), (B.2)-(B.4), (B.5’), (B.6), (B.7’):

$\begin{matrix} \int_{\Omega_{I}} C_{\Delta}(t)dx= \\ =\left(\int_{\Omega_{I}} P_{\Delta}(0)dx+\frac{\rho_{PT}}{d_{T}}\int_{\Omega_{I}} T_{\Delta}(0)dx\right)\left[\begin{matrix} \frac{[\rho_{CM}-d_{CM}C_{eq}]\rho_{M}}{d_{M}(0)d_{Cm}} \\ -C_{eq}[\mu-\rho_{M}]\left(t-\frac{1}{d_{Cm}m_{eq}}\right) \end{matrix}\right] \\ -\frac{C_{eq}}{m_{eq}}\left(\int_{\Omega_{I}} m_{\Delta}(0)dx-\frac{m_{eq}[\mu-\rho_{M}]\rho_{PT}\int_{\Omega_{I}} T_{\Delta}(0)dx}{d_{T}^{2}}\right)+O(\exp[-\delta t]) \end{matrix}$ (B.11)

$\int_{\Omega_{I}} P_{\Delta}(t)dx=\int_{\Omega_{I}} P_{\Delta}(0)dx+\frac{\rho_{PT}}{d_{T}}\int_{\Omega_{I}} T_{\Delta}(0)dx+O(\exp[-\delta t])$ (B.12)

$\begin{matrix} \int_{\Omega_{I}} m_{\Delta}(t)dx=\int_{\Omega_{I}} m_{\Delta}(0)dx \\ +m_{eq}[\mu-\rho_{M}]\left\{\begin{matrix} t\left(\int_{\Omega_{I}} P_{\Delta}(0)dx+\frac{\rho_{PT}}{d_{T}}\int_{\Omega_{I}} T_{\Delta}(0)dx\right) \\ -\frac{\rho_{PT}}{d_{T}^{2}}\int_{\Omega_{I}} T_{\Delta}(0)dx \end{matrix}\right\}\text{ }+O(\exp[-\delta t]) \end{matrix}$ (B.13)

$\int_{\Omega_{I}} M_{\Delta}(t)dx=\frac{\rho_{M}m_{eq}\left(\int_{\Omega_{I}} P_{\Delta}(0)dx+\frac{\rho_{PT}}{d_{T}}\int_{\Omega_{I}} T_{\Delta}(0)dx\right)}{d_{M}(0)}+O(\exp[-\delta t])$ (B.14)

$\int_{\Omega_{I}} \eta_{\Delta}(t)dx=\rho_{in}(0)L_{0}\frac{\rho_{M}m_{eq}\left(\int_{\Omega_{I}} P_{\Delta}(0)dx+\frac{\rho_{PT}}{d_{T}}\int_{\Omega_{I}} T_{\Delta}(0)dx\right)}{d_{M}(0)^{2}}+O(\exp[-\delta t])$ (B.15)

$\begin{matrix} \int_{\Omega_{I}} N_{\Delta}(t)dx=\int_{\Omega_{I}} N_{\Delta}(0)dx+\int_{\Omega_{I}} \eta_{\Delta}(0)dx \\ +\left[\begin{matrix} d_{TT}-\frac{\rho_{in}(0)L_{0}\rho_{M}m_{eq}\rho_{PT}}{d_{T}^{2}d_{M}(0)} \\ -\frac{d_{MM}\rho_{M}m_{eq}\rho_{PT}}{d_{T}^{2}} \end{matrix}\right]\int_{\Omega_{I}} T_{\Delta}(0)dx+\left[\frac{\rho_{in}(0)L_{0}}{d_{M}(0)}+d_{MM}\right]\int_{\Omega_{I}} M_{\Delta}(0)dx \\ \left(\int_{\Omega_{I}} P_{\Delta}(0)dx+\frac{\rho_{PT}}{d_{T}}\int_{\Omega_{I}} T_{\Delta}(0)dx\right)\left[\begin{matrix} \frac{\rho_{in}(0)L_{0}\rho_{M}m_{eq}\left(t-\frac{2}{d_{M}(0)}\right)}{d_{M}(0)} \\ +d_{MM}\rho_{M}m_{eq}\left(t-\frac{1}{d_{M}(0)}\right) \end{matrix}\right] \\ +O(\exp[-\delta t]) \end{matrix}$ (B.16)

for some $\delta>0$, so that this shows that $C_{\Delta},P_{\Delta},m_{\Delta},M_{\Delta},\eta_{\Delta},N_{\Delta}$ do not generally decay to 0 as $t\to\infty$. We now show that $T_{\Delta}$ does decay to 0 as $t\to\infty$, at least in $L^{2}$. Multiplying (2.38) by $T_{\Delta}$ and integrating over the intima, $\Omega_{I}$, we have by (41) again that:

$\frac{1}{2}\frac{\partial}{\partial t}\int_{\Omega_{I}} T_{\Delta}^{2}dx=-d_{T}\int_{\Omega_{I}} T_{\Delta}^{2}dx-D_{T}\int_{\Omega_{I}} |\nabla T_{\Delta}|^{2}dx$ (B.17)

From this $||T_{\Delta}(t)||_{2}^{2}=\int_{\Omega_{I}} T_{\Delta}(t)^{2}dx\leq\int_{\Omega_{I}} T_{\Delta}(0)^{2}dx\exp\left[-2d_{T}t\right]$ so that if $d_{T}>0$ then:

$\lim_{t\to\infty}||T_{\Delta}(t)||_{2}=0$ (B.18)

[ is the standard $L^{2}$ norm.] From Table S2 $d_{T}=6.9\text{ x }10^{-7}s^{-1}>0$ so again this condition is always likely to be satisfied.
