# Supplementary material for: A Model of Cardiovascular Disease Giving a Plausible Mechanism for the Effect of Fractionated Low-Dose Ionizing Radiation Exposure
Source: PLoS Comput Biol. 2009 Oct 23;5(10):e1000539. doi: 10.1371/journal.pcbi.1000539 (PMC2759077; doi:10.1371/journal.pcbi.1000539)
Supplement: Table S1 — Candidate molecules for variables in the model. (0.04 MB DOC) [file pcbi.1000539.s002.doc]

**Table S1. Candidate molecules for variables in the model.**

| Variable | Description | Compound |
| --- | --- | --- |
| $r$ | Free radical concentration | nitrous oxide (NO), ascorbate free radical [27] |
| $v$ | Anti-oxidant concentration | vitamin C, vitamin E |
| $C$ | Chemo-attractant | MCP-1 [16], IL-6, IL-8 [28, 29] |
| $P$ | Monocyte proliferation factor | interferon-γ [30], macrophage CSF [31] |
