# Supplementary material for: A Model of Cardiovascular Disease Giving a Plausible Mechanism for the Effect of Fractionated Low-Dose Ionizing Radiation Exposure
Source: PLoS Comput Biol. 2009 Oct 23;5(10):e1000539. doi: 10.1371/journal.pcbi.1000539 (PMC2759077; doi:10.1371/journal.pcbi.1000539)
Supplement: Table S2 — Parameters and estimated values. (0.35 MB DOC) [file pcbi.1000539.s003.doc]

**Table S2. Parameters and estimated values.**

| Variable | Description | Value | Equation | References |
| --- | --- | --- | --- | --- |
| $k_{L_{i}}$ | Rate (per unit radical concentration) of oxidation of LDL (with oxidation state $i>1$) | 3 x 10^-6^ M^-1^ s^-1^ | A.1, A.2, A.5 | Ingold *et al.* [32], Cobbold *et al.* [22] |
|  | Rate (per unit radical concentration) of oxidation of nearly fully oxidized LDL (with oxidation state $i=1$) | 6 x 10^-11^ M^-1^ s^-1^ | A.1, A.2, A.5 | Ingold *et al.* [32], Cobbold *et al.* [22] |
| $k_{H_{i}}$ | Rate (per unit radical concentration) of oxidation of HDL (with oxidation state $i>1$) | 4 x 10^-7^ M^-1^ s^-1^ | A.3, A.4, A.5 | Cobbold *et al.* [22] |
|  | Rate (per unit radical concentration) of oxidation of nearly fully oxidized HDL (with oxidation state $i=1$) | 7.9 x 10^-12^ M^-1^ s^-1^ | A.3, A.4, A.5 | Cobbold *et al.* [22] |
| $\omega_{L_{i}}$ | Rates (per unit anti-oxidant concentration) of de-oxidation of LDL (with oxidation state $i$) | 1.55 x 10^-6^ M^-1^ s^-1^ | A.1, A.2, A.6 | Packer *et al.* [33], Jialal *et al.* [34] |
| $\omega_{H_{i}}$ | Rates (per unit anti-oxidant concentration) of de-oxidation of HDL (with oxidation state $i$) | 2.0 x 10^-7^ M^-1^ s^-1^ | A.3, A.4, A.6 | Packer *et al.* [33], Jialal *et al.* [34] |
| $\rho_{in}()$ | Function describing rate of conversion per unit of fully oxidized LDL and per unit macrophage concentration to lipid-bound LDL (i.e. to LDL ingested into macrophages)(function of bound lipid to macrophage ratio $\eta/M$) | - | A.19, 7 | McKay *et al.* [1] |
| $\rho_{in,0}=\rho_{in}(0)$ | Bound lipid ingestion rate at zero lipid concentration | 5.3 x 10^-10^ cell^-1^ ml s^-1^ | A.21, A.23 | Crouse *et al.* [35], Schwegler *et al.* [36], Zhao *et al.* [26]^a^ |
| $\rho_{in,high}$ | Limiting bound lipid ingestion rate at high lipid concentration | 2.7 x 10^-10^ cell^-1^ ml s^-1^ | A.23 | Crouse *et al.* [35], Schwegler *et al.* [36], Zhao *et al.* [26]^a^ |
| $R_{1}$ | Denominator scaling constant in bound lipid ingestion rate function, $\rho_{in}\left(\eta/M\right)$ | ? | A.21 |  |
| $R_{3}$ | Exponential scaling constant in bound lipid ingestion rate function, $\rho_{in}\left(\eta/M\right)$ | 2.1 x 10^2^ cell M ^-1^ | A.21, A.23 | Crouse *et al.* [35], Schwegler *et al.* [36], Zhao *et al.* [26]^a^ |
| $d_{ox}$ | Rate of reduction of free radical as a result of oxidation reactions with vitamin E and HDL | ? | A.11 |  |
| $d_{antox}$ | Rate of reduction of anti-oxidant as a result of anti-oxidation reactions with vitamin E and LDL or HDL | ? | A.12 |  |
| $N_{\max,L}$ | Maximum number of oxidized LDL species (maximum number of vitamin E molecules+1) | 7-16 | A.7, A.11, A.12, 1, 2 | Esterbauer *et al.* [5], Cobbold *et al.* [22] |
| $N_{\max,H}$ | Maximum number of oxidized HDL species (maximum number of vitamin E molecules+1) | 2 | A.9, A.11, A.12 | Cobbold *et al.* [22] |
| $\tau$ | Repopulation rate of endothelial cells | 4.6 x 10^-6^ s^-1^ | A.13, 1 | Sachs & Brenner [37] |
| $\iota_{L_{i}}$ | Rates of damage of endothelial cells per unit of LDL concentration with oxidation status $i$ | 0 for $i>0$ 6.1 x 10^1^ ml M^-1^ s^-1^ for $i=0$ | A.13, A.14, 1, 2 | Crouse *et al.* [35], Thomas *et al.* [38], Liu *et al.* [39], Steffen *et al.* [40]^b^ |
| $E_{0ss}$ | Target EC concentration | 1.3 x 10^9^ cells ml^-1^ | A.13, 1 | McGeachie [41] |
| $\rho_{CE}$ | Chemo-attractant production rates per unit concentration of damaged ECs | 5.7 x 10^-24^ M cell^-1^ s^-1^ | A.15, 3 | Filler *et al.* [42]^c^: MCP-1 |
| $\rho_{CM}$ | Chemo-attractant production rates per unit concentration of macrophages | 2.0 x 10^-25^ M cell^-1^ s^-1^ | A.15, 3 | Bosco *et al.* [43], Yoo *et al.* [44]^d^: MCP-1 |
| $\rho_{CT}$ | Chemo-attractant production rates per unit concentration of T-cells | 0 M cell^-1^ s^-1^ | A.15, 3 | Villiger *et al.* [45]: IL6, Lukacs *et al.* [46]: MCP-1 |
| $\rho_{CS}$ | Chemo-attractant production rates per unit concentration of VSMCs | ? M cell^-1^ s^-1^ | A.15 |  |
| $\rho_{PT}$ | Monocyte proliferation factor production rate per unit of T-lymphocyte concentration | 9.1 x 10^-25^ M cell^-1^ s^-1^ | A.16, 4 | Tang *et al.* [47], Knowles [48], Frétier *et al.* [49]^e^: M-CSF |
| $\rho_{M}$ | Monocyte→macrophage proliferation/conversion rate | 3.5 x 10^9^ ml M^-1^ s^-1^ | A.17, A.18, 5, 6 | Becker *et al.* [50]^f^: M-CSF |
| $\mu$ | Monocyte proliferation rate per unit of proliferation factor | 3.5 x 10^9^ ml M^-1^ s^-1^ | A.17, 5 | Becker *et al.* [50]^f^: M-CSF |
| $d_{E}$ | Death/degradation rates of damaged ECs | 1 x 10^-4^ s^-1^ | A.14, 2 | Dimmeler *et al.* [51] |
| $d_{S}$ | Death/degradation rates of VSMCs | 1 x 10^-5^ s^-1^ | A.25 | McKay *et al.* [1] |
| $d_{T}$ | Death/degradation rates of T-cells | 6.9 x 10^-7^ s^-1^ | A.20, A.27, 8, 9 | Asquith *et al.* [52] |
| $T_{L}$ | Lumenal T-lymphocyte concentration | 2.5 x 10^6^ cells ml ^-1^ | 15, 18 | Hanson [53] |
| $m_{L}$ | Lumenal monocyte concentration | 3.0 x 10^5^ cells ml ^-1^ | 12, 17 | Hanson [53] |
| $d_{M}()$ | Function describing rate of release of bound lipid due to macrophage death (per unit of bound lipid concentration and per macrophage concentration) (function of bound lipid to macrophage ratio $\eta/M$) | - | A.18, A.19, 6, 7, 9 | McKay *et al.* [1] |
| $d_{M,0}=d_{M}(0)$ | Underlying macrophage mortality rate | 9.3 x 10^-7^ s^-1^ | A.22, A.24 | Crouse *et al.* [35], Schwegler *et al.* [36], Kellner-Weibel *et al.* [54]^g^ |
| $R_{2}$ | Scaling constant in macrophage death rate function, $d_{M}\left(\eta/M\right)$ | 7.7 x 10^13^ cell M^-1^s^-1^ | A.22, A.24 | Crouse *et al.* [35], Schwegler *et al.* [36], Kellner-Weibel *et al.* [54]^g^ |
| $J_{m}(C,m_{L})$ | Function determining influx of monocytes as function of MCP-1 concentration, lumenal monocyte concentration | - | 12, 17 |  |
| $\beta_{m,0}$ | Parameter in function $J_{m}(C,m_{L})$ determining constant influx of monocytes as function of lumenal monocyte concentration | 1.7 x 10^8^ m^-2^ | 17 | Takaku *et al.* [55]^h^ |
| $\beta_{m,1}$ | Parameter in function $J_{m}(C,m_{L})$ determining influx of monocytes as function of MCP-1 concentration, lumenal monocyte concentration | 2.5 x 10^13^ M^-1^ ml m^-2^ | 17 | Takaku *et al.* [55]^h^ |
| $C_{Tm}$ | Threshold chemo-attractant level for monocyte flux into intima | ? M ml^-1^ | 17 |  |
| $J_{T}(C,T_{L})$ | Function determining influx of T-lymphocytes as function of MCP-1 concentration, lumenal T-lymphocyte concentration | - | 15, 18 |  |
| $\beta_{T,0}$ | Parameter in function $J_{T}(C,T_{L})$ determining constant influx of T-lymphocytes as function of lumenal T-lymphocyte concentration | 7.3 x 10^7^ m^-2^ | 18 | Klouche *et al.* [56], Takaku *et al.* [55]^i^ |
| $\beta_{T,1}$ | Parameter in function $J_{T}(C,T_{L})$ determining influx of T-lymphocytes as function of MCP-1 concentration, lumenal T-lymphocyte concentration | 8.9 x 10^12^ M^-1^ ml m^-2^ | 18 | Klouche *et al.* [56], Takaku *et al.* [55]^i^ |
| $C_{TT}$ | Threshold chemo-attractant level for T-lymphocyte flux into intima | ? M ml^-1^ | 18 |  |
| $d_{CM}$ | Chemo-attractant degradation rate per unit macrophage concentration | 1.3 x 10^-5^ ml cell^-1^ s^-1^ | A.15, 3 | Han *et al.* [57], Seet *et al.* [58], Cullen *et al.* [59]: MCP-1^j^ |
| $d_{CT}$ | Chemo-attractant degradation rate per unit T-cell concentration | 1.3 x 10^-5^ ml cell^-1^ s^-1^ | A.15, 3 | Han *et al.* [57], Seet *et al.* [58], Cullen *et al.* [59]: MCP-1^j^ |
| $d_{Cm}$ | Chemo-attractant degradation rate per unit monocyte concentration | 1.3 x 10^-5^ ml cell^-1^ s^-1^ | A.15, 3 | Han *et al.* [57], Seet *et al.* [58], Cullen *et al.* [59]: MCP-1^j^ |
| $n_{1}$ | Power of $\left(\eta/M\right)$ in bound lipid ingestion rate function, $\rho_{in}\left(\eta/M\right)$ | ? | A.21 |  |
| $n_{2}$ | Power of $\left(\eta/M\right)$ in macrophage death rate function, $d_{M}\left(\eta/M\right)$ | 1 | A.22 |  |
| $\rho_{G}$ | Collagen augmentation rate per unit of VSMC concentration | ? | A.26 |  |
| $J_{mech}$ | Degradation rate of VSMC due to mechanical stress | ? | A.25 |  |
| $d_{GT}$ | Destruction/degradation rates of collagen per unit T-cell concentration | ? | A.26 |  |
| $d_{GM}$ | Destruction/degradation rates of collagen per unit macrophage concentration | ? | A.26 |  |
| $d_{MM}$ | Ratio of rate of augmentation of necrotic core by dying macrophages (multiplier of $d_{M}(\eta/M)$) | 1.5 x 10^-8^ M cell^-1^ | A.27, 9 | Krombach *et al.* [60]^k^ |
| $d_{SS}$ | Multiplier of VSMC for volume of necrotic core | 1 M cell^-1^ | A.27 | McKay *et al.* [1] |
| $d_{TT}$ | Ratio of rate of augmentation of necrotic core by T-cell death | 2.1 x 10^-10^ M cell^-1^ | A.27, 9 | Cheung *et al.* [61]^l^ |
| $D_{L_{i}}$ | Rate of diffusion of LDL (with oxidation state $i$) in intima | 5.6 x 10^-12^ m^2^ s^-1^ | A.1, A.2 | Crouse *et al.* [35]^m^ |
| $D_{H_{i}}$ | Rate of diffusion of HDL (with oxidation state $i$) in intima | 1.9 x 10^-11^ m^2^ s^-1^ | A.3, A.4 | Atmeh [62]^m^ |
| $D_{r}$ | Rate of diffusion of radicals in intima | ? m^2^ s^-1^ | A.5 |  |
| $D_{v}$ | Rate of diffusion of anti-oxidants in intima | ? m^2^ s^-1^ | A.6 |  |
| $D_{l,L_{i}}$ | Rate of diffusion of LDL (with oxidation state $i$) in lumen | ? m^2^ s^-1^ | A.7, A.8 |  |
| $D_{l,H_{i}}$ | Rate of diffusion of HDL (with oxidation state $i$) in lumen | ? m^2^ s^-1^ | A.9, A.10 |  |
| $D_{l,r}$ | Rate of diffusion of radicals in lumen | ? m^2^ s^-1^ | A.11 |  |
| $D_{l,v}$ | Rate of diffusion of anti-oxidants in lumen | ? m^2^ s^-1^ | A.12 |  |
| $D_{C}$ | Rate of diffusion of chemo-attractant | 3.9 x 10^-11^ m^2^ s^-1^ | A.15, 3 | Chandrasekhar [63]^m^: MCP-1^n^ |
| $D_{P}$ | Rate of diffusion of monocyte proliferation factor | 1.8 x 10^-11^ m^2^ s^-1^ | A.16, 4 | Chandrasekhar [63]^m^, Suzu *et al.* [64]: M-CSF |
| $D_{m}$ | Rate of diffusion of monocytes | 3.0 x 10^-15^ m^2^ s^-1^ | A.17, 5 | Chandrasekhar [63]^m^, Lund *et al.* [65], Shin *et al.* [66] |
| $D_{M}$ | Rate of diffusion of macrophages | 2.9 x 10^-15^ m^2^ s^-1^ | A.18, A.19, 6 | Chandrasekhar [63]^m^, Krombach *et al.* [60], Shin *et al.* [66]^o^ |
| $D_{T}$ | Rate of diffusion of T-cells | 6.2 x 10^-15^ m^2^ s^-1^ | A.20, 8 | Chandrasekhar [63]^m^, Cheung *et al.* [61] |
| $D_{S}$ | Rate of diffusion of VSMCs | ? m^2^ s^-1^ | A.25 |  |
| $\chi_{m}(C)$ | Chemotactic factor associated with monocytes | 3.0 x 10^0^ m^2^ ml s^-1^ M^-1^ | A.17, 5 | Lauffenburger *et al.* [67] |
| $\chi_{M}(C)$ | Chemotactic factor associated with macrophages | 3.0 x 10^0^ m^2^ ml s^-1^ M^-1^ | A.18, 6, 7 | Lauffenburger *et al.* [67] |
| $\chi_{T}(C)$ | Chemotactic factor associated with T-cells | 3.0 x 10^0^ m^2^ ml s^-1^ M^-1^ | A.20, 8 | Lauffenburger *et al.* [67] |
| $\chi_{S}(C)$ | Chemotactic factor associated with VSMCs | ? m^2^ ml s^-1^ M^-1^ | A.25 |  |

^a^based on fitting (via non-linear least squares) to data in Figure 3 of Zhao *et al.* [26], integrating d(*η*/*M*)/dt = *ρ*_in_(*η*/*M*)*L*_0_ (derived *via* equation (7)), using the functional form (A.23), and using LDL molecular weight of 2.93 x 10^6^ [35] and estimate of 136 pg protein/cell [36].

^b^geometric mean of figures derived from papers of Thomas *et al.* [38], Liu *et al.* [39], Steffen *et al.* [40], using LDL molecular weight of 2.93 x 10^6^ [35] to convert to molar concentrations.

^c^figures derived from paper of Filler *et al.* [42], and using also estimate (*via* website <http://www.exalpha.com/pdfs/X1243C.pdf>) of MCP-1 molecular weight of 8.7 kD to convert to molar concentration.

^d^geometric mean of figures derived from papers of Yoo *et al.* [44] (IL-18 stimulated and unstimulated cells), Bosco *et al.* [43] (normal and hypoxic cells), and using also estimate (*via* website <http://www.exalpha.com/pdfs/X1243C.pdf>) of MCP-1 molecular weight of 8.7 kD to convert to molar concentration.

^e^figure derived from paper of Frétier *et al.* [49], assuming that d*P*/dt = *ρ_PT_T* - *kP* and using estimate of half-life of MCP-1, 50 mins [47] to estimate *k*; conversion factor of 1 activity unit M-CSF = 0.44 fM [48].

^f^assuming that d*m*/dt = *μmT* - *ρ_M_Pm* , d*M*/dt = *ρ_M_Pm* – *km* and using also estimate of 1 activity unit of M-CSF = 0.44 fM, and M-CSF molecular weight of 70 kD [48].

^g^based on LDL molecular weight of 2.93 x 10^6^ [35] and estimate of 136 pg protein/cell [36].

^h^based on fitting linked differential equations *J_m_*(*C*,*m*) = d*m*/dt = *D_m_*[*β*_0_,*_m_*+*β*_1_,*_m_C*]*m_L_*, d*m_L_*/dt = -*D_m_*[*β*_0_,*_m_*+*β*_1_,*_m_C*]*m_L_* to data in Figure 4 of Takaku *et al.* [55].

^i^based on scaling of differential equation *J_T_*(*C*,T) = d*T*/dt = *D_m_*[*β*_0_,*_T_*+*β*_1_,*_T_C*]*m_T_* from one for monocytes fitted to data in Figure 4 of Takaku *et al.* [55], using ratios of control and 25 μg ml^-1^ E-LDL percentage transmigrations for T-lymphocytes and monocytes from data in Figure 4 of Klouche *et al.* [56].

^j^based on a 1-point binding concentration, *K_d_*, estimated by Cullen *et al.* [59] as 1.2 x 10^-24^ M ml^-1^ cell^-1^ (average of control levels in Figures 1, 2), an estimated 5000 CCR2 receptors for MCP-1 per cell [57] and an ORFZ- NZ2-CPB-MCP-1 dissociation rate of 1.86 x 10^-3^ s^-1^ [58].

^k^figure derived from macrophage diameter given in Krombach *et al.* [60].

^l^figure derived from T-lymphocyte volume given in Cheung *et al.* [61].

^m^based on formula for diffusion coefficient *D* = *kT*/(6*πηr*) [63], where *k* = Boltzmann constant = 1.38065 x 10^-23^ J K^-1^, *T* = temperature = 310 K, *η* = viscosity = 0.01 Pa s^-1^ [66], *r*=radius of molecule; this was adapted in some cases from values on the website [www.math.ubc.ca/~ais/website/status/diffuse.html](http://www.math.ubc.ca/~ais/website/status/diffuse.html), scaling by the inverse cube root of molecular weight relative to the value for IL-6.

^n^figure derived from IL-6 diffusion coefficient and molecular weight (from website [www.math.ubc.ca/~ais/website/status/diffuse.html](http://www.math.ubc.ca/~ais/website/status/diffuse.html)), and estimate (via website <http://www.exalpha.com/pdfs/X1243C.pdf>) of MCP-1 molecular weight of 8.7 kD, using inverse cube-root scaling.

^o^geometric mean of figures derived from papers of Krombach *et al.* [60], Shin *et al.* [66] and website [www.math.ubc.ca/~ais/website/status/diffuse.html](http://www.math.ubc.ca/~ais/website/status/diffuse.html).
