# Supplementary material for: A Model of Cardiovascular Disease Giving a Plausible Mechanism for the Effect of Fractionated Low-Dose Ionizing Radiation Exposure
Source: PLoS Comput Biol. 2009 Oct 23;5(10):e1000539. doi: 10.1371/journal.pcbi.1000539 (PMC2759077; doi:10.1371/journal.pcbi.1000539)
Supplement: Table S3 — Values for perturbed species. (0.07 MB DOC) [file pcbi.1000539.s004.doc]

**Table S3. Values for perturbed species.**

Values assumed for simulations of averaged perturbed species, using equations (42)-(48), as given in Figures 1-8.

| Variable | Description | Value | Reference/source |
| --- | --- | --- | --- |
| $L_{0}$ | Oxidised LDL concentration in intima | 3.1 x 10^-10^ M ml^-1^ | Napoli *et al.* [68], Bing *et al.* [69]^a^ |
| $L_{1}$ | Unoxidised LDL concentration in intima | 4.9 x 10^-6^ M ml^-1^ | Henry *et al.* [70]^b^ |
| $C_{eq}$ | Equilibrium value of chemo-attractant in intima | 7.9 x 10^-15^ M ml^-1^ | Cannon *et al.* [71]^c^ |
| $SD[C_{eq}]$ | Standard deviation of equilibrium value of chemo-attractant in intima | 9.3 x 10^-15^ M ml^-1^ | Cannon *et al.* [71]^c^ |
| $m_{eq}$ | Equilibrium value of monocytes in intima | 4.0 x 10^3^ cells ml^-1^ | Navab *et al.* [72], Lichtman *et al.* [73]^d^ |
| $m_{\Delta}(0)$ | Perturbation of monocytes in intima | -2.4 x 10^3^ cells ml^-1^ Gy^-1^ | Gordon [74], Navab *et al.* [72], Lichtman *et al.* [73]^e^ |
| $E_{0,eq}$ | Normal equilibrium endothelial cell concentration | 1.3 x 10^9^ cells ml^-1^ | McGeachie [41]^f^ |
| $E_{0,\Delta}$ | Perturbation of normal endothelial cells | -8.4 x 10^8^ cells ml^-1^ Gy^-1^ | Deschavanne & Fertil [75], McGeachie [41]^g^ |
| $E_{1,eq}$ | Damaged equilibrium endothelial cell concentration | 7.1 x 10^7^ cells ml^-1^ | Eq. (29) |
| $E_{1,\Delta}$ | Perturbation of damaged endothelial cells | -4.5 x 10^7^ cells ml^-1^ Gy^-1^ | Deschavanne & Fertil [75], Eq. (29)^g^ |
|  | Arterial endothelial cell cross sectional area | 2.8 x 10^-7^ m^2^ | McGeachie [41], Leung *et al.* [76]^h^ |
|  | Cross sectional area of intima apart from endothelial cell layer | 8.5 x 10^-6^ m^2^ | Terry *et al.* [77], Leung *et al.* [76]^i^ |

^a^geometric mean of figures derived from papers of Napoli *et al.* [68] and Bing *et al.* [69].

^b^mean of values for men and women in Table 1 of Henry *et al.* [70].

^c^figures derived from paper of Cannon *et al.* [71], and using also estimate (*via* website <http://www.exalpha.com/pdfs/X1243C.pdf>) of MCP-1 molecular weight of 8.7 kD to convert to molar concentration.

^d^derived from monocyte concentration in blood from Lichtman *et al.* [73], and assuming 1% of these migrate to intima, as suggested by Figure 4 of Navab *et al.* [72].

^e^assuming 1% (via Navab *et al.* [72]) of value for monocytes in blood from Lichtman *et al.* [73] and assuming a cellular radio-sensitivity of 0.599 Gy^-1^ (=1/(1.67 Gy)) (Table 2 Gordon [74]).

^f^assuming endothelial cells are approximately rectangular cuboid with dimensions 15 μm x 5 μm x 10 μm, packed in a layer 10 μm high [41].

^g^derived from equilibrium value assuming a cellular radio-sensitivity of 0.633 Gy^-1^ (=1/(1.58 Gy)) (Table 2 Deschavanne and Fertil [75]).

^h^assuming endothelial cells are packed in a layer 10 μm high [41], and assuming a coronary artery diameter of 4.49 x 10^-3^ m [76] implies an endothelial cell cross section of π x 0.00001 x 4.49 x 10^-3^ m^2^.

^i^assuming intima media thickness of 1.08 mm [77] and coronary arterial diameter of 4.49 x 10^-3^ m [76] implies an intimal cross section of π x [(0.00449+0.00108)^2^-0.00449^2^]/4 m^2^.
